# Integrating macroeconomic and technical indicators into forecasting the stock market: a hybrid approach for efficient feature selection

**DOI:** 10.1038/s41598-026-67696-3

**Published:** 2026-09-02

**Authors:** Aya Nabil, Sherif Barakat, Ahmed Aboelfetouh, Sara Elhishi

**Affiliations:** 1 Information Systems Department, Faculty of Computers and Information, Mansoura University, Mansoura, Egypt; 2 Delta Higher Institute for Management and Accounting Information Systems, Mansoura, Egypt; 3 Faculty of Computer Studies, Arab Open University of Egypt, Cairo, Egypt

**Keywords:** Stock market prediction, Technical indicators, Macroeconomic indicators, Feature selection, Deep learning, Statistical hypothesis test, Economic significance, Trading profitability, Economics, Economics, Finance, Finance, Mathematics and computing

## Abstract

Stock market prediction is a significant research topic in the financial sector and has been widely investigated by researchers and investors. Forecasting stock markets is challenging because of the nonlinearity, volatility, and dynamics of the data. Additionally, stock markets are affected by various internal and external factors. While most previous studies relied on historical prices and technical indicators (TIs), they ignored the influence of overall economic factors on stock markets. In contrast, this study introduces a robust framework to predict daily log returns by leveraging a combined dataset comprising historical data, TIs, and macroeconomic data, including gold and oil prices, the volatility index, the dollar index, the interest rate, the 10-year Treasury yield, the term spread, and the dividend yield. Moreover, we propose a hybrid feature selection (FS) approach that combines filter and wrapper methods, unlike in previous studies, which relied primarily on a single FS approach or neglected it entirely. The dataset represents diverse sectors and firm sizes, covering five companies: Apple (AAPL Inc.), Exxon Mobil Corporation (XOM), Goldman Sachs (GS), Pfizer Inc. (PFE), and Ford Motor Company (F). We apply a hybrid evaluation approach that incorporates 5-fold time series cross-validation (TSCV) with a holdout test set to evaluate the efficiency of the proposed models. The results revealed that despite modest improvements in statistical metrics, FS significantly enhanced the economic performance of the trading strategy, as demonstrated by better returns and Sharpe ratios. The results show that deep learning (DL) models that combine macroeconomic data and the FS process, in addition to historical data and TIs, achieved higher trading returns and produced superior risk-adjusted performance, although they demonstrated slightly higher forecast errors than traditional models. This confirms that statistical accuracy alone is insufficient for evaluating financial forecasting models. This work highlights the benefits of the proposed framework for predicting stock returns across different markets, offering significant insights for financial analysts.

## Introduction

Predicting stock markets is a challenging yet essential topic for traders, investors, and researchers, as it enables them to understand market trends^1^. The stock market plays an essential role in today’s global economy. Predicting stock market trends, prices, movements, fluctuations, etc., is a major challenge in financial markets because of the chaotic, complex, dynamic, and nonparametric nature of the data^2^. Additionally, different internal and external factors affect stock market trends, prices, returns, and movements. The internal factors include historical stock prices and investor sentiment, and the external factors include macroeconomic data, international agreements, and political stability^3,4^.

Stock market prediction (SMP) is a significant topic in the financial sector. It provides a comprehensive approach that involves analyzing historical stock prices and their movements to forecast future prices^5^. SMP is a major issue in time series analysis, which involves analyzing previous stock data to anticipate future movements, thereby providing benefits to individuals, governments, and corporations^6^.

Investors and traders use different tools and methods to maximize profits and mitigate risks. They try to anticipate the stock market and forecast future stock prices to decide whether to sell their current stocks or purchase other stocks. Stock traders can make significant profits by accurately forecasting stock price trends at the right time. Consequently, SMP is an essential element of stock traders’ decision-making process^7,8^.

Traditional statistical models assume linearity and stationarity and therefore struggle to capture the nonlinear dynamics of financial data. Machine Learning (ML) approaches relaxed these assumptions and achieved better predictive performance. Conventional feedforward neural networks further improve the nonlinear modeling capability. However, they still have a limited ability to capture long-term dependencies and the complex sequential nature of financial time series^9^. Consequently, advanced DL architectures have been increasingly adopted for stock forecasting because of their superior ability to learn nonlinear and sequential patterns.

Different DL models, such as recurrent neural networks (RNNs), long short-term memory networks (LSTMs)^10–12^, gated recurrent units (GRUs)^13^, convolution neural networks (CNNs)^14,15^, and other hybrid models, including CNN-LSTM, GRU-LSTM, and CNN-GRU^16–18^, have been developed for predicting stock prices. Additionally, the authors of^19^ provide a comprehensive comparison of traditional statistical techniques and ML and DL approaches for SMP. The results revealed that the DL models outperformed the statistical and ML techniques.

The prediction of stock market prices, trends, and movements involves two main approaches. The first approach uses open, high, low, close, and volume (OHLCV) as the main feature. In contrast, the second approach combines additional significant features, along with historical stock data, such as TIs, macroeconomic data, and other factors, to improve predictive performance^20^.

The high dimensionality of stock market data is a significant issue in SMP. It comprises many features, which increase the computational complexity and lead to model overfitting. FS techniques can address this challenge by identifying and extracting the most informative features that significantly affect prediction results while discarding irrelevant features.

Most previous studies have relied on historical baseline features (OHLCVs) and TIs while overlooking the impact of overall economic factors. In contrast, in this study, we not only address the challenge of predicting stock returns as a regression task but also incorporate TIs, macroeconomic indicators, and volatility features as significant factors in analyzing stock markets. Moreover, unlike most existing studies that neglected FS entirely or relied on a single approach, we introduce a hybrid FS approach to overcome these limitations.

We introduce a robust framework to forecast daily log returns by integrating OHLCV, TIs, and macroeconomic data, including the volatility index, oil and gold prices, the dollar index, the 10-year Treasury yield, the term spread, and the dividend yield, with advanced DL models to capture market fluctuations effectively. Additionally, we develop an efficient FS approach. We execute it through a hybrid approach that integrates filter and wrapper methods to select the most significant features.

The proposed models include LSTM, bidirectional LSTM (Bi-LSTM), GRU, and bidirectional GRU (Bi-GRU), as well as hybrid modes such as GRU-LSTM and CNN-GRU. The GRU-LSTM combines the advantages of the GRU and LSTM models to capture both short-term dependencies and long-term fluctuations in time series data. Additionally, the CNN-GRU combines the strengths of CNNs and GRUs to capture long-term dependencies, reduce complexity, and increase efficiency.

This study examines whether better prediction accuracy means better trading results. The answer is no. The results indicated that a model with lower prediction errors is not necessarily more profitable, whereas one with slightly higher errors may produce better returns by correctly predicting the direction of price movements, which determine profitability. Therefore, we evaluate the proposed models in terms of both statistical accuracy and trading performance. Empirical results show that, despite the linear regression (LR) and random forest (RF) outperforming the Bi-GRU in terms of statistical accuracy, the Bi-GRU delivered superior trading returns and risk-adjusted performance across five stocks. This confirms that economic significance matters more than forecast error in financial forecasting.

The purpose of this work is to assist stakeholders in financial markets, including investors, policymakers, traders, and analysts, by providing robust forecasting models and facilitating the decision-making process.

The novelties of our research study are as follows:

- Stock price data for five companies, namely, Apple, XOM, GS, PFE, and F, are collected, and various steps for the data preprocessing stage are performed to achieve better and more accurate results.
- By incorporating OHLCV, TIs, volatility features, and macroeconomic features into a unified dataset, stock return forecasting can be enhanced, in contrast to most previous studies that relied on OHLCV and TIs.
- An efficient FS approach that integrates filter and wrapper methods is proposed to extract the most important features, unlike in previous studies that relied on a single approach, and the impact of this hybrid approach on the efficiency of different DL models, such as LSTM, GRU, Bi-LSTM, Bi-GRU, GRU-LSTM, and CNN-GRU, is evaluated.
- A hybrid evaluation approach that combines 5-fold TSCV with a holdout test set is proposed, unlike most existing studies that rely on the holdout method.
- The efficiency and robustness of the proposed models are evaluated via standard forecasting metrics such as the mean squared error (MSE), root mean squared error (RMSE), mean absolute error (MAE), and directional accuracy (DA), as well as a trading simulation analysis.
- An ablation study is conducted on the basis of the best-performing model to analyze the contribution of each component within the proposed framework.
- The Wilcoxon signed-rank test, the Friedman test, the Finner post hoc test, and the Diebold–Mariano (DM) test are applied as statistical hypothesis tests to ensure the reliability and validity of the proposed work.
- Develop the Shapley Additive exPlanations (SHAP) technique, using the best-performing model across all stocks to interpret the impact of each feature on predicting stock returns.

The remainder of this paper is structured as follows: Sect.  2 presents relevant studies on SMP; Sect.  3 describes the proposed framework; Sect.  4 provides the findings, comparisons, and discussion. Finally, Sect.  5 discusses the conclusions and explains future directions.

## Related work

This section covers an overview of the relevant studies on SMP. Predicting stock prices plays a significant role in financial markets. Different studies have been conducted to predict stock prices, trends, returns, and movements over the next trading days via various techniques. Table 1 below summarizes the previous work considered in this study.

**Table 1:** Analysis of SMP.

Study	Types of features	FS technique	Prediction methods	Dataset/Stocks	Evaluation metrics
^11^	Basic features, TIs	Not applicable	LSTM	NASDAQ and NYSE	MSE, RMSE, MAE, MAPE
^13^	Basic features	Not applicable	LSTM, GRU	4 stocks from NSE, NASDAQ	RMSE, MAE, R2
^29^	Basic features, TIs	Not applicable	LSTM-GRU	TSE	MSE
^30^	Basic features	Not applicable	ARMA-RNN	6 stocks from NSE	MAE RMSE
^31^	Historical cryptocurrency data	Not applicable	GRU, LSTM, Bi-LSTM	Bitcoin, Litecoin, Ethereum	RMSE, MAPE
^32^	Basic features, TIs	Not applicable	LSTM, GRU, CNN	Nepal Stock Exchange (NEPSE)	RMSE, MAPE, R2
^33^	Basic features, TIs	Not applicable	CNN, LSTM, CNN-LSTM	21 stocks from NSE	RMSE, MAE, MAPE, R2
^34^	Basic features	Not applicable	Vanilla LSTM, Stacked LSTM, Bi-LSTM, ConvLSTM, Encoder-Decoder-LSTM	NSE	RMSE, MAE, R2
^35^	Basic features	Not applicable	Bi-LSTM	Apple	MSE
^36^	Basic features, TIs	Not applicable	CNN, LSTM, CNN-LSTM, DT, LR, RF	-	Variance, R2 Score, Max Error
^37^	Basic features, TIs	Not applicable	ANN, RNN, LSTM, DT, FR, AdaBoost, XGBoost, Gradient Boosting	Iranian stock market	MAPE, MAE, RMSE, MSE
^38^	Basic features, TIs	Not applicable	single-layer LSTM, three-layer LSTM, CNN-LSTM, three-layer Bi-LSTM	Istanbul Stock Exchange (ISE)	MAPE, MAE, RMSE, MSE, R2
^39^	Basic features, TIs	Not applicable	LSTM, XGBoost, LSTM-XGBoost	International stock markets such as DAX, MASI, HKEX, CAC 40, NASDAQ, and FTSE 250	MAPE, MAE, RMSE, R2
^40^	Basic features	Not applicable	LSTM, RF, XGBoost, ANN, SVM, AdaBoost	TCS	MAE, RMSE, R2
^41^	Basic features	Not applicable	RNN, LSTM, GRU, Bi-LSTM, Bi-GRU	TCS	MAPE
^42^	Basic features, TIs, and macroeconomic data	Correlation heatmap	LSTM	S&P 500	MAPE, RMSE, R2
^43^	Basic features, TIs	Not applicable	ELM, LSTM, ANN, XGBoost	NASDAQ, NYSE	MSE, MAE, RMSE, SMAPE
^44^	Basic features, TIs	PCC	BLS	4 stocks from the Shanghai Stock Exchange	MSE, MAE, MAPE, R2
^45^	Basic features	PCA, RFE IG	-	Netflix, Amazon, Domino’s Pizza	Accuracy
^46^	Basic features, TIs	RFE	SVM, RF, ANN	Chinese A-share market	Accuracy, AUC
^47^	Basic features, TIs	RFE DT, RF	LSTM	Apple, Microsoft, Facebook	MSE, MAE, RMSE
^48^	Basic features, TIs	SFS, SBE	Linear, Lasso Ridge Regression, DT, KNN, MLP, RF, SVM, Gradient Boosting, AdaBoost	Apple	MSE, RMSE, MAE, MAPE
^49^	Basic features, TIs	SBE	LSTM	NIFTY 50	MSE, RMSE, MAE
^50^	Basic features, TIs	PCA	RF, Bagging, AdaBoost, XGBoost	8 stocks from NYSE, NASDAQ, NSE	Accuracy, precision, F1-score, AUC
^51^	TIs	PCA	Gaussian Naïve Bayes	7 stocks from NYSE, NASDAQ, NSE	Accuracy, F1-score, AUC
^52^	Basic features, TIs	PCA LASSO	LSTM GRU	Shanghai Composite Index	MAE, RMSE, MSE
^53^	Basic features, TIs, VIX	LASSO	GRU	CSI 300	MAE, RMSE, MAPE, R2
^54^	Basic features, TIs	PCA	LSTM-TI	Standard & Poor’s 500, NASDAQ, Apple (AAPL)	MAE, RMSE
^55^	macroeconomic data	Not applicable	Frequency-domain	U.S. stock market	Economic measures
^56^	Basic features, macroeconomic data	Not applicable	ANN, DNN	U.S. stock market	RMSE, MAE
^57^	VIX, EPU, GPR	Not applicable	Wavelet-based connectedness analysis	Commodity and financial markets	Dynamic connectedness measures
^58^	Term spread, Dividend Yield, Inflation	Not applicable	Nonparametric predictive regression	U.S. stock market	Sharp ratio, Economic gains
^59^	Real-time macroeconomic variables	Not applicable	Predictive regression, ML models	U.S. Treasury bond market	Economic measures
^60^	Macroeconomic expectations	Not applicable	Predictive asset pricing models	U.S. financial markets	Economic gains

### From linear models to ML and DL for predicting stock prices

Different parametric models, such as autoregressive moving average (ARMA), autoregressive integrated moving average (ARIMA), and generalized autoregressive conditional heteroskedasticity (GARCH), have been frequently used for SMP^21–25^. However, traditional techniques fail to identify and extract the nonlinear behavior of financial markets and achieve satisfactory predictive results when large volumes of data with complexity and high volatility are used^10^. Different ML models, including decision trees (DTs), RF, support vector machines (SVMs)^26^, and LR^27^, have been developed for stock price prediction and enhanced performance, but fail to capture sequential dependencies in financial data. Various DL models have been proposed to address this challenge by efficiently capturing nonlinear patterns, which are typically used to anticipate different datasets and extract hidden patterns and useful information for predicting stock market trends^27,28^. However, most previous studies used stock prices, not log returns, as the prediction target, which raises nonstationary concerns that compromise the reliability of the results. In this section, we discuss previous studies that used different models to predict stock prices.

Chandar^11^ proposed an LSTM model to forecast the stock closing price using two stock exchanges, NASDAQ and NYSE. Different TIs were combined with historical stock data. The LSTM was compared with ARIMA-RNN, RNN, and LSTM-TI. This model outperformed the others, achieving MAE and RMSE values of 0.017 and 0.022, respectively, whereas Chang et al. ^13^ evaluated the efficiency of LSTM and GRU to predict stock prices of different stocks, i.e., AAPL, MSFT, and AMZN, obtained from the NASDAQ and NYSE stock exchanges. They considered only OHLCV in their work. The GRU outperformed the LSTM model across all stocks.

Farhadi et al.^29^ combined the strengths of both LSTM and GRU to capture short and long dependencies. The dataset was fetched from the Tehran Stock Exchange (TSE) for eight stocks. These datasets included the OHLCV features and TIs. The LSTM-GRU model achieved better performance, reducing MSE by 3% compared to other models. Rather et al.^30^, a robust hybrid model to forecast stock returns using ARMA as a linear model combined with RNN as a nonlinear model. The results confirmed that the hybrid model produced promising performance, with an MAE of 0.017 and an RMSE of 0.031.

Hamayel and Owda^31^ explored the efficiency of different DL models, including LSTM, GRU, and Bi-LSTM, for forecasting cryptocurrency prices. The GRU model surpassed the others; in contrast, the Bi-LSTM achieved the worst performance. Pokhrel et al.^32^ LSTM, GRU, and CNN were developed for predicting stock closing prices. The input sequences used in this study included OHLCV and TIs. The LSTM model achieved better results and surpassed both GRU and CNN models.

Singh et al.^33^ integrated the advantages of CNN and LSTM, employing stock market data and TIs to detect complex fluctuations using different stocks from the NSE. Their research revealed the effectiveness of the CNN-LSTM in forecasting stock prices. The CNN-LSTM model surpassed the others across various evaluation metrics, whereas Pattanayak et al.^34^ used various LSTM models, including Stacked LSTM, Bi-LSTM, ConvLSTM, and Encoder-Decoder-LSTM using single and multiple layers. The dataset was gathered from the NSE for different companies. The single-layer LSTM outperformed the other LSTM models.

Han and Fu^35^ proposed the Bi-LSTM model to predict Apple Inc.‘s closing prices using only its historical price data. Additionally, its performance was evaluated using MSE. The Bi-LSTM model outperformed the LSTM model, especially for handling the volatility. Zhao et al.^36^ developed a hybrid CNN-LSTM model using OHLCV and TIs to predict stock closing prices. The efficiency of the CNN-LSTM was compared with DT, LR, RF, LSTM, and CNN. The proposed model achieved better results and surpassed the others.

Nabipour et al.^37^ evaluated the performance of ANN, RNN, LSTM, DT, RF, AdaBoost, Gradient Boosting, and XGBoost to predict future prices of different stocks using TIs as the input data. The LSTM achieved more reliable results than other models. Konur et al.^38^ employed various DL models, including a single-layer LSTM, a three-layer LSTM, a CNN-LSTM, and a three-layer Bi-LSTM, using historical stock data from five companies, along with other TIs. The dataset was gathered from the Istanbul Stock Exchange (ISE) in Turkey. The findings confirmed that the single-layer LSTM surpassed the other models.

Oukhouya et al.^39^ developed ML models, including LSTM, XGBoost, and a hybrid LSTM-XGBoost, using daily stock price data from various markets. The LSTM-XGBoost model improved the accuracy of closing price forecasting, outperforming both the LSTM and XGBoost models, whereas Gupta et al.^40^ developed LSTM, RF, XGBoost, ANN, SVM, and AdaBoost models to predict stock prices. The stock price data was gathered from Tata Consultancy Services (TCS). The LSTM achieved the highest accuracy (0.89), surpassing the others.

Kalidindi et al.^41^ evaluated the performance of RNN, LSTM, GRU, Bi-LSTM, and Bi-GRU using only OHLCV features. The GRU and LSTM models outperformed the RNN model. Additionally, GRU and Bi-GRU outperformed the other models. Bhandari et al.^42^ proposed single- and multilayer LSTM models to forecast the closing price of the S&P 500 index. This study combines OHLCV, TIs, and other macroeconomic data to capture fluctuations in stock markets. It was shown that the single LSTM achieved superior performance compared to multiple LSTM layers.

Pashaei and Gürül^43^ introduced the Extreme Learning Machine (ELM) with the fusion of multiple TIs for forecasting stock closing prices using different datasets, including AAPL, ORCL, MSFT, GS, and IBM. They used MSE, MAE, and RMSE to assess the effectiveness of the proposed model. They compared the ELM model with ANN, LSTM, and XGBoost. The ELM model produced significant results and surpassed other models across all metrics and datasets.

### FS approaches for predicting stock prices

SMP remains a complex and challenging task because of its nonlinear dynamics and multifactorial indicators. It involves identifying the most relevant features and using them to achieve better results. FS can enhance model performance by reducing irrelevant features, mitigating overfitting, and reducing computational complexity. In this section, we discuss studies that have used various FS methods for forecasting stock prices.

In^44–54^, different FS approaches were applied to forecast stock closing prices and trends. Li et al.^44^ proposed a framework based on Pearson’s correlation coefficient (PCC) to identify the most important feature, using OHLCV and TIs as the main features and a Broad Learning System (BLS) model for predicting stock closing prices. The PCC-BLS model outperforms the other traditional models. Paramita and Winata^45^ evaluated the efficacy of three approaches—principal component analysis (PCA), recursive feature elimination (RFE), and information gain (IG)—for forecasting stock market trends. They used different datasets, including Netflix (NTFX), Amazon (AMZN), and Domino’s Pizza (DPZ). It was observed that the RFE surpassed the other approaches in forecasting stock market value, achieving high accuracy.

Yuan et al.^46^ employed the RFE to extract the importance scores of all features. They selected the top 80% of the features. They proposed three models, namely, SVM, RF, and ANN, to forecast the direction of stock returns. Botunac et al.^47^ employed RFE, DT, and RF to predict the stock closing prices of Apple, Facebook, and Microsoft. Moreover, predictions were made using the LSTM model. The findings revealed that specific features influence LSTM model performance, underscoring the importance of selecting the most important features before building predictive models.

Moodi et al.^48^ examined the performance of different ML models, including LR, LASSO regression, DT, KNN, MLP, SVR, gradient boosting, AdaBoost regression, and RF, using AAPL stock data, along with TIs. Sequential forward selection (SFS) and sequential backward elimination (SBE) were used to evaluate their performance in identifying the most important features and predicting model results. All the proposed models demonstrated improved performance when the SFS and SBE approaches were used. Jafar et al.^49^ compared the efficiency of LSTM and SBE-LSTM using daily stock price data from the NIFTY 50 index to predict the closing price. The proposed models were compared with ARIMA, RF, and multiple regression. Compared with the other models, the SBE-LSTM model achieved higher accuracy (95%).

Ampomah et al.^50^ and Ampomah et al.^51^ employed PCA using OHLCV and TIs for forecasting stock price movements. They combined PCA with various ML classifiers and reported that PCA-based models outperformed non-PCA models. Gao et al.^52^ compared the efficiency of LSTM and a GRU using OHLCV, TIs, and financial data. They applied PCA and LASSO feature selection methods and evaluated the performance of the proposed forecasting models. The LSTM and GRU models achieved better performance with LASSO than with PCA.

Fang et al.^53^ proposed a GRU model combined with LASSO to increase the training speed and a volatility index to mitigate prediction errors as VIX-LASSO-GRU to forecast intraday stock prices in China. The proposed model outperforms the LSTM and GRU models in terms of both accuracy and speed. They highlighted the advantages of the volatility index and the FS process in trading. Gao and Chai^54^ introduced an LSTM model combined with various TIs (LSTM-TI) to forecast stock closing prices using PCA to reduce the number of features. This method achieved a lower MAE value of 0.02 and resulted in an RMSE value of 0.03 when AAPL stock was used, demonstrating the importance of incorporating OHLCV with TIs.

As illustrated above, traditional studies rely on a single FS technique, leading to overreliance on specific elements or the exclusion of informative ones. By combining multiple FS approaches, this study can identify hidden patterns and trends in stock market data from various perspectives, thereby avoiding biases that result from the use of a single approach. In this way, we can improve the performance of the models and FS.

### Return predictability and economic relevance

The financial economics literature consistently argues that return series, not prices, are theoretically appropriate and stationary forecasting targets. The following studies highlight a growing trend in the use of macroeconomic predictors for return-based forecasting.

Dai et al.^55^ employed wavelet analysis on the S&P 500 returns in conjunction with the ML technique to construct a composite macroeconomic index from the 14 predictors given by Welch and Goyal (2008). Their findings indicated that this composite index surpassed each macroeconomic predictor, with the predictive ability being most significant during times of high economic uncertainty. Additionally, the term spread is the most influential return predictor.

Wang^56^ employed SHAP-based interpretability to assess the relative significance of firm characteristics and macroeconomic variables in forecasting various stock returns via neural network models. The results indicated that a predictor’s importance varies by the return measure: macroeconomic variables, especially those that characterize investor sentiment, such as VIX, were the leading predictors for raw or excess returns, whereas firm-specific traits (momentum and trading turnover) were major factors for forecasting abnormal returns after adjusting for asset pricing factors. SHAP analysis revealed that the VIX is the most important predictor.

Chen et al.^57^ analyzed the dynamics of connectedness among economic policy uncertainty (EPU), the VIX, and geopolitical risk (GPR) and commodity/financial markets. They revealed that the VIX had the strongest and most stable predictor and consistent impact on market connectedness, whereas EPU and GPR revealed more complicated, time-varying effects that changed from positive to negative over time.

Cheng et al.^58^ presented a multiphase nonparametric prediction model that used dividend yield as one of the most significant predictors and other macroeconomic variables, such as term spread, the treasury bill rate, and the inflation rate, to forecast stock returns. The results indicated that the proposed model achieved better results than the other baseline models. They revealed that the dividend yield is a robust return predictor.

Huang et al.^59^ explored the predictability of U.S. bond returns by employing real-time macroeconomic data and reported that different macroeconomic indicators, especially yield spreads and interest rates, significantly predict investment. They showed that using real-time macroeconomic data enhanced the accuracy of forecasts and led to substantial investment profitability. Across the stock and bond markets, the term spread is the most consistent predictor.

Deng et al.^60^ examined the ability of macroeconomic expectations to forecast asset returns. They combined various expectations, such as interest rates, inflation rates, economic growth, and monetary policy, to construct forecasting models. They reported that compared with traditional forecasting models, incorporating these variables significantly enhanced the accuracy of return predictions.

All of the above studies highlight the importance of incorporating macroeconomic variables into forecasting models.

### Research gaps and contributions

Four gaps persist in the literature despite growing interest in the use of DL for financial forecasting:

1. Non-stationary price levels are used as a forecasting target. To address this gap, we forecast log returns.
2. Depending on a single FS technique, robustness is limited. The present study addresses this challenge by proposing a hybrid FS approach that integrates filter and wrapper methods.
3. Using standard forecasting metrics and ignoring economic evaluation. To address this gap, we evaluate the proposed models by conducting a trading analysis (strategy returns and Sharpe ratio) that compares them against a simple buy-and-hold strategy.
4. Lack of interpretability. To address this gap, we apply SHAP analysis to determine the most influential factors for each stock. The results are linked to established economic theories, including monetary policy transmission, risk premia, and technical trading behavior.

### Proposed framework

**Fig. 1:**
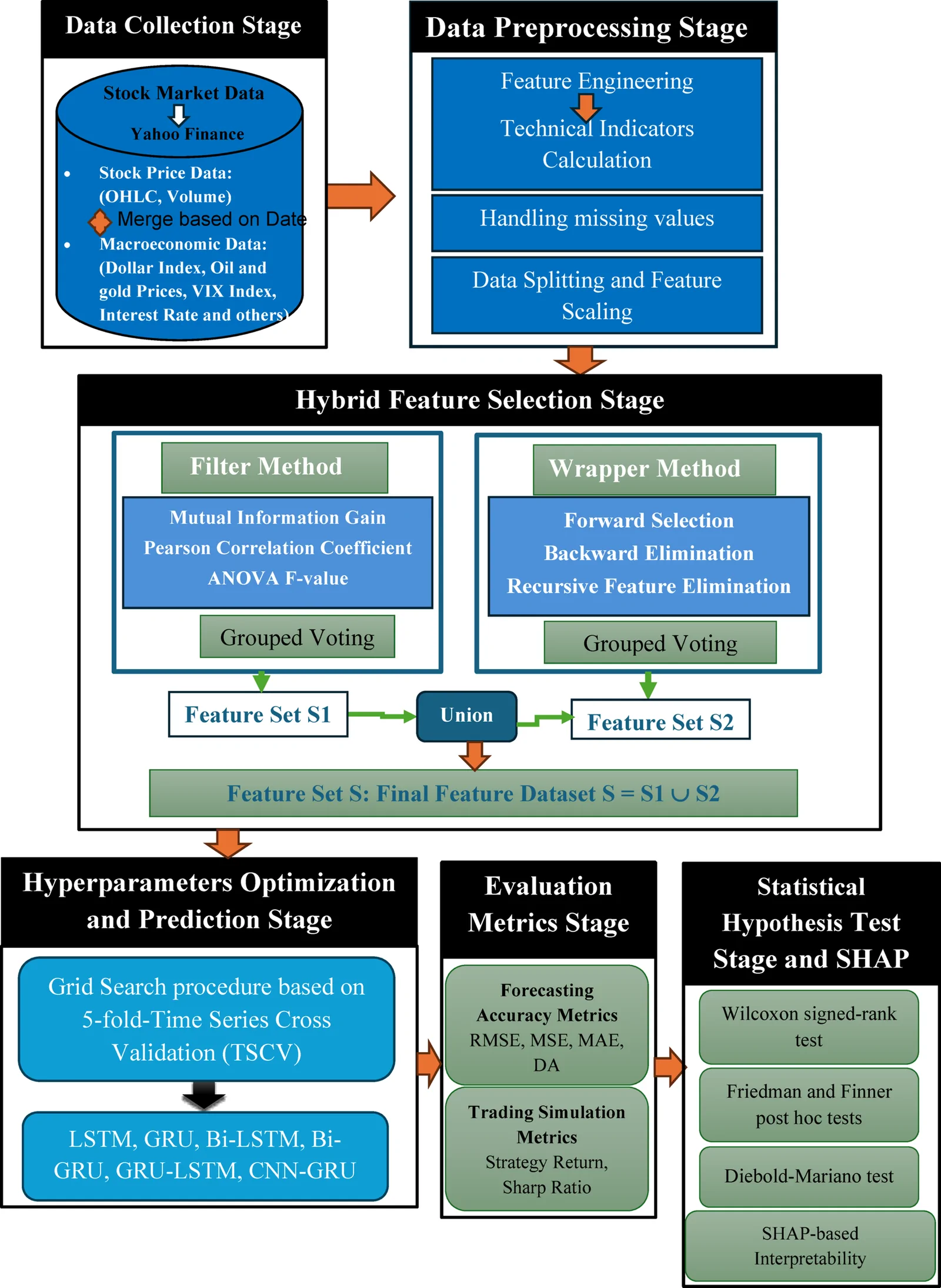
Proposed framework for predicting daily log returns.

The main purpose of this paper is to propose different DL models to predict daily log returns instead of stock prices because return series are considered more stationary than raw price series and are the main focus of predictability research in financial economics. We aim to forecast stock returns via a combination of historical stock price data, different TIs, and macroeconomic data. The proposed framework consists of different stages: data collection, data preprocessing, hybrid FS approach, model validation and prediction, statistical and economic evaluation, statistical hypothesis testing, and SHAP-based interpretability. These stages are described below. The proposed framework for predicting daily log returns is presented in Fig. 1.

Additionally, we conduct some types of trading analyses, such as strategy total return and the Sharpe ratio, that compare against a simple buy-and-hold benchmark.

### Data collection stage

The historical stock price data and macroeconomic indicators were collected from the Yahoo Finance API. In this study, we selected companies from the two largest stock exchanges, the NASDAQ and the New York Stock Exchange (NYSE), spanning the period from January 2005 to May 30, 2024, encompassing 4887 trading days. This period covers a full economic cycle: the 2008 financial crisis, the following recovery and bull-run market, and the COVID-19 turmoil, rather than beginning at the recession trough. The selected companies represent diverse sectors and firm sizes, including AAPL (Technology, Mega Cap), XOM (Energy, Mega Cap), GS (Financial Services, Large Cap), Pfizer (Healthcare, Large Cap), and Ford (Automotive Manufacturing, Mid Cap), enabling the evaluation of the proposed model across different industries. Each data sample includes various features, such as date, open, high, low, close, and volume (OHLCV).

**Fig. 2:**
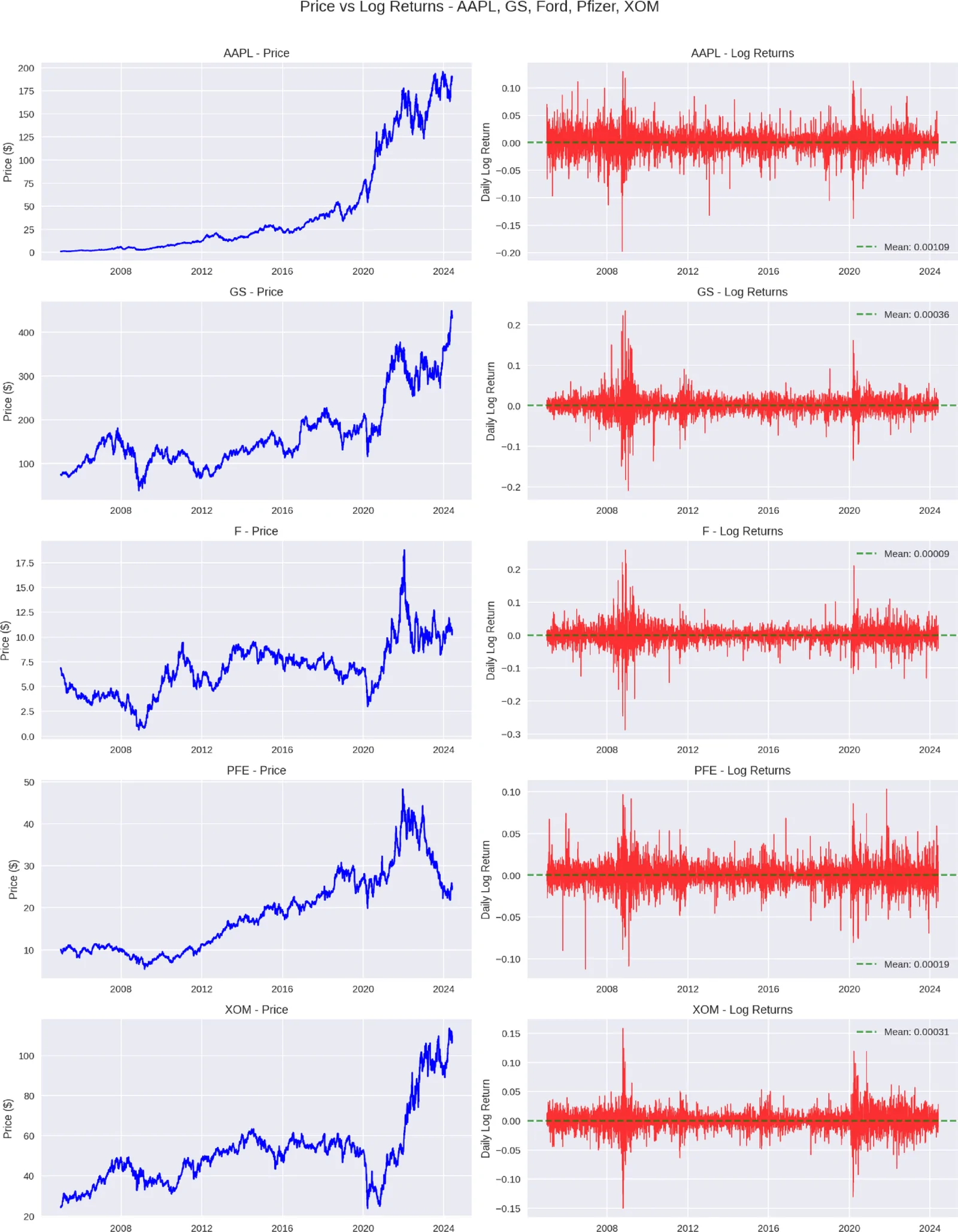
Stock prices vs. Log returns for all stocks.

The stock price series and daily log returns of five stocks are compared in Fig. 2. In contrast to price series that are nonstationary and exhibit strong trends over time, the log returns fluctuate around near zero, and they do not exhibit a persistent trend, confirming their suitability as the prediction target in this study.

### Data preprocessing stage

Data preprocessing is a significant step in ML and data mining. It is the process of converting raw data into a suitable, efficient format that can handle errors, support more effective model learning, produce more precise predictions, and enhance model performance.

In our study, we used various steps to improve the quality of the collected datasets, including target variable (log returns) construction, feature engineering, handling missing values, data splitting, and feature scaling. These steps are presented below.

### Target construction

In this study, the target variable is the daily log return, which is computed as follows:

1 $$ {\mathrm{r}}_{{\mathrm{t}}} = \log \frac{{{\mathrm{P}}_{{\mathrm{t}}} }}{{{\mathrm{P}}_{{{\mathrm{t}} - 1}} }} $$

where $\:{\mathrm{P}}_{\mathrm{t}}$ is the closing price at time t and $\:{\mathrm{P}}_{\mathrm{t}-1}$ is the closing price at time t-1.

### Feature engineering

It is the process of generating new features from existing historical raw data. It is a very important step that helps models learn more effectively, extract hidden patterns, and achieve more accurate predictions^61^.

In our study, we aim to forecast the log return on the next trading day. Using only OHLCV features as inputs to predict stock closing prices is insufficient because of stock market fluctuations, price movements, global conditions, and other factors. To address this problem, we extract different TIs from OHLCV data.

TIs are essential tools that facilitate the analysis of stock market trends, prices, and movements, enabling more accurate trading decisions. They are a very important step in providing insights into stock market behavior, price movements, volatility, and fluctuations. Additionally, they play important roles in analyzing stock markets, extracting meaningful hidden patterns, and forecasting future trends^62–64^.

TIs are classified into different categories. These categories are trend indicators, momentum indicators, volatility indicators, and volume indicators^6,29^. They provide a summary representation of stock market trends in time series data.

The dataset includes both internal and external factors. The internal factors include different values of TIs and volatility features, such as simple moving average (SMA), exponential moving average (EMA), relative strength index (RSI), moving average convergence divergence (MACD), Williams %R, rate of change (ROC), stochastic %K, and %D, Volume_MA_20, Price_to_SMA_21, Volatility_20d, and intraday volatility, which are calculated from OHLCV prices. Moreover, a wide range of external factors, such as the dollar index (DXY), oil and gold prices, and the volatility index (VIX), were added as macroeconomic data and the global market. Monetary policy conditions and the term structure of interest rates were represented by the interest rate (Treasury Bill Rate), 10-year Treasury yield, and term spread. Furthermore, dividend yield was used to measure the valuation of stocks and their expected returns as a firm-level financial indicator. These variables were chosen on the basis of their relevance to the financial economics literature.

The selected features are listed in Table 2. First, we download historical stock prices and macroeconomic data via the Yahoo Finance API in Python. We then calculate different TIs and finally merge the macroeconomic dataset with historical stock data and TIs by date.

**Table 2:** Features used.

Category	Variable	Formula (if applicable)	Type	Source
Historical Price Data	Open (O)	Opening Price	Price	Yahoo Finance
	High (H)	Highest Price		
	Low (L)	Lowest Price		
	Close (C)	Closing Price		
	Volume	Volume of transactions	Volume	
TIs	SMA	$\:\frac{\sum\:_{\mathrm{i}=1}^{\mathrm{n}}{\mathrm{C}}_{\mathrm{i}}}{\mathrm{n}}\:\:\:\:\:\:\:\mathrm{n}=7,\:21\:$	Trend	Calculated
	EMA	$\:\frac{2}{\mathrm{n}+1}\left({\mathrm{C}}_{\mathrm{t}\:}-{\mathrm{E}\mathrm{M}\mathrm{A}}_{\mathrm{t}-1}\:\right)\:+{\mathrm{E}\mathrm{M}\mathrm{A}}_{\mathrm{t}-1}\:\:\:\:\:\mathrm{n}=7,\:21\:$	Trend	
	RSI	$\:100-\left[\frac{100}{1+\mathrm{r}\mathrm{e}\mathrm{l}\mathrm{a}\mathrm{t}\mathrm{i}\mathrm{v}\mathrm{e}\:\mathrm{s}\mathrm{t}\mathrm{r}\mathrm{e}\mathrm{n}\mathrm{g}\mathrm{t}\mathrm{h}}\right]$	Momentum	
	MACD	$\:\mathrm{E}\mathrm{M}\mathrm{A}\left(12\right)-\mathrm{E}\mathrm{M}\mathrm{A}\left(26\right)$	Trend	
	MACD Signal	*EMA (MACD*,* 9)*	Trend	
	MACD Histogram	*MACD-Signal*	Momentum	
	Stochastic %K	$\:100\:\frac{{\mathrm{C}}_{\mathrm{t}}-{\mathrm{C}}_{\mathrm{L}\left(\mathrm{n}\right)}}{{\mathrm{C}}_{\mathrm{H}\left(\mathrm{n}\right)}-{\mathrm{C}}_{\mathrm{L}\left(\mathrm{n}\right)}}\:\:\:\:\:\:\:\:\mathrm{n}=14\:$	Momentum	
	Stochastic %D	$\:\mathrm{E}\mathrm{M}\mathrm{A}\:(\mathrm{\%}\mathrm{K},\:3)$	Momentum	
	ROC	$\:\:\left(\frac{{\mathrm{P}\mathrm{r}\mathrm{i}\mathrm{c}\mathrm{e}}_{\mathrm{t}}}{{\mathrm{P}\mathrm{r}\mathrm{i}\mathrm{c}\mathrm{e}}_{\mathrm{t}-\mathrm{n}}}\right)\mathrm{*}100$	Momentum	
	Williams %R	$\:\frac{({\mathrm{H}}_{\mathrm{t}}-{\mathrm{C}}_{\mathrm{t}})}{{\mathrm{H}}_{\mathrm{t}}-{\mathrm{L}}_{\mathrm{t}}}\mathrm{*}100$	Momentum	
	Volume_MA_20	$\:\frac{\sum\:_{\mathrm{i}=1}^{20}{\mathrm{V}\mathrm{o}\mathrm{l}\mathrm{u}\mathrm{m}\mathrm{e}}_{\mathrm{t}-\mathrm{i}}}{20}$	Volume	
	Price_to_SMA_21	$\:\frac{{\mathrm{C}}_{\mathrm{t}}}{{\mathrm{S}\mathrm{M}\mathrm{A}}_{21}}$	Trend	
	Volatility_20d	$\:{\upsigma\:}\left({\mathrm{R}\mathrm{e}\mathrm{t}\mathrm{u}\mathrm{r}\mathrm{n}}_{1\mathrm{d}},20\right)\:\times\:\sqrt{252}$	Volatility	
	Intraday_Volatility	$\:\frac{{\mathrm{H}}_{\mathrm{t}}-{\mathrm{L}}_{\mathrm{t}}}{{\mathrm{O}}_{\mathrm{t}}}$	Volatility	
Macroeconomic Data	Dollar_Index	US Dollar Index (DXY)	Currency	Yahoo Finance
	VIX_Index	Volatility Index/Fear Index	Risk	
	Gold_Price	Gold Price (USD per troy ounce)	Commodity	
	Oil_Price	Crude Oil Price (WTI)	Commodity	
	Interest_Rate	13-Week Treasury Bill Rate	Short-term Interest Rate	
	Treasury_10Y	10-Year Treasury Bill Rate	Long-term Interest Rate	
	Term Spread	Term Spread (10Y-13 W)	Yield curve	Calculated
	Dividend Yield	Trailing 12-Month	Trailing Dividend	Firm-derived

### Handling missing values

Missing values naturally appear after feature engineering. TIs based on rolling windows, such as the SMA, EMA, RSI, and ROC, cannot be computed until a minimum number of preceding observations are available. It is a critical stage in data analysis, especially in data preprocessing. Handling missing values in the dataset can improve data quality, prevent modeling errors, and enhance model performance. Different strategies are used to handle missing values. The mean imputation destroys the time series structure of the variables. Therefore, in this study, we used the bidirectional second-order polynomial interpolation strategy. This approach preserves the data’s temporal continuity instead of introducing artificial discontinuities.

### Data splitting

To prevent data leakage and to avoid look-ahead bias, we first divided the entire dataset into 80% for training and 20% for testing to isolate the holdout test set before any data scaling, feature selection, or model training.

### Feature scaling

It is an important step in data preprocessing, ensuring that all the input features are scaled consistently. Feature scaling can reduce bias, accelerate model learning, facilitate faster convergence, and improve model performance^65^.

We normalized the stock returns via the standard scaler. It scales all features via the following formula:

2 $$\:{\mathrm{X}}_{\mathrm{s}\mathrm{c}\mathrm{a}\mathrm{l}\mathrm{e}\mathrm{d}}=\frac{\mathrm{X}-{\upmu\:}}{{\upsigma\:}}$$

where $\:\mathrm{X}$ is the original value and $\:{\upmu\:}$ and $\:{\upsigma\:}$ are the average and standard deviation, respectively.

### Hybrid FS stage

FS is the process of selecting the most important features and ignoring irrelevant features. It is an essential stage that not only reduces irrelevant features, overfitting, and computational costs but also improves model performance and accelerates training time^66–68^.

The filter and wrapper methods are distinct approaches to feature selection. The filter method, as a statistical technique, assesses feature significance by using metrics such as correlation or information gain without ML models. Features are extracted on the basis of their correlation with the target. They ignore feature interactions while being computationally fast and efficient^69^.

In contrast, the wrapper method employs an ML model to evaluate the quality of different feature subsets, aiming to maximize performance while considering feature interactions. However, they are prone to overfitting and may be computationally expensive^69^.

The hybrid FS approach is performed only on the training set to avoid look-ahead bias, while the holdout test set is reserved for the final assessment, using the selected feature.

We propose a hybrid approach that combines the filter and wrapper methods for FS. Combining both methods is essential to ensure that we retain only the top-ranked features, capture robust features to mitigate bias introduced by any single method, and enhance the efficiency and performance of the proposed models.

First, we employed three filter methods, namely, the PCC, mutual information gain (MIG), and analysis of variance (ANOVA), to measure both linear and nonlinear relationships between features. The PCC measures the linear relationship between features and the target (log return), whereas the MIG measures both linear and nonlinear relationships among features. ANOVA is a statistical method used to evaluate differences among groups of data. It evaluates the importance of differences by examining the variance within the means of the data groups^70^.

We extract the top-ranked features obtained from the PCC, MIG, and ANOVA methods. Afterward, we apply a grouped voting strategy, where the features extracted by at least two methods within the filter group are retained, ensuring that only the most important features are selected, denoted S1.

Next, we employed three wrapper methods: SFS, SBE, and RFE. SFS begins with no features. Afterward, it adds features one at a time, selecting the feature that improves the model’s performance. SBE begins with all features and then gradually removes the least important features one at a time^49,71^. RFE is a combination of the SBE method. It begins with all features and then recursively removes the least important features on the basis of the model’s performance until it reaches the desired number of features^69^. SFS and SBE are trained via linear regression (LR), whereas RFE is trained via random forest (RF). RF provides a ranking score for each feature in the entire dataset. The ranking scores are used to select the relevant features and eliminate the least important ones.

We extract the top-ranked features generated from the SFS, SBE, and RFE methods. Afterward, we apply the same grouped voting strategy to retain the most significant features within the wrapper group, denoted as S2.

After six FS methods, which are based on two categories—filter and wrapper—are used to generate different feature subsets, we combine them to obtain the final feature set $\:\mathrm{S}=\mathrm{S}1\:\cup\:\mathrm{S}2\:\:$based on the top-ranked features.

Combining three filter methods and three wrapper methods for FS has several benefits for identifying strong, shared features. This hybrid method increases the likelihood of identifying the most important features by combining the strengths of different methods. This strategy improves the overall efficacy of FS by combining the best features from the filter and wrapper methods. This can enhance model accuracy and robustness.

## Experimental settings

The experiments were implemented and trained on a Google Colab environment and the Python programming language. To provide frameworks for developing, implementing, and evaluating the proposed models, we used a range of libraries, including yfinance, Pandas, NumPy, SciPy, Matplotlib, Scikit-Learn, TensorFlow, and Keras.

### Model training and hyperparameter optimization

We employed a hybrid evaluation approach that combines TSCV with a holdout test set to evaluate the efficiency and robustness of the proposed models. Hyperparameter optimization was performed using a 5-fold TSCV with a grid search on the training set to select the optimal hyperparameters for the DL models (such as units, dropout, and batch size) and for the RF (such as n_estimators and max_depth).

The hyperparameter search space is described below in Table 3, with the optimal configuration for each model. We choose the combination with the lowest average validation loss across all folds. After the best hyperparameters were determined, each model was retrained on the whole training set before being tested once during the isolated holdout period, enabling it to learn from all the training data rather than the limited subsets utilized during cross-validation. Finally, we computed all the forecasting accuracy and trading simulation metrics. This approach ensures that hyperparameter tuning and final assessment are strictly separated while still thoroughly exploiting the training data to produce the final models.

**Table 3:** Hyperparameter search space and their selected optimal values for each model.

Model	Search Space	Optimal Configuration
LSTM	Units: 100,200 Dropout: 0.2, 0.3 Batch Size: 32, 64	LSTM (100), Dropout (0.2), Dense (1)
GRU		GRU (100), Dropout (0.2), Dense (1)
Bi-LSTM		Bi-LSTM (100), Dropout (0.2), Dense (100), Dense (1)
Bi-GRU		Bi-GRU (200), Dropout (0.3), Dense (100), Dense (1)
GRU-LSTM	Units: 128, 160 Dropout: 0.2, 0.3 Batch Size: 32, 64	GRU (128), LSTM (128), Dropout (0.3), Dense (64), Dense (1)
CNN-GRU		Conv1D (64, k = 3), Max-pooling (2), GRU (128), GRU (128), Dropout (0.2), Dense (1)
RF	n_estimators: 100, 200 max_depth: 3, 5	n_estimators: 100 max_depth: 5

Experimental Settings: Epochs = 50, Optimizer=Adam (0.001), Batch Size = 64.

Activation Functions: Recurrent layers: tanh, Dense Layers: ReLU, Output layer: linear.

CV is an essential technique in ML used to evaluate model performance on distinct subsets of data. The dataset is randomly divided into training and testing sets. K-fold CV is the most common type of CV technique. It randomly shuffles the data and divides it into k equal folds. Afterward, one of the folds is used for testing, and the model is trained on the other folds (K-1). This process is repeated k times; each time, a different fold is used as the test set. The results from each fold are subsequently combined by averaging to assess the overall performance^72^.

This standard K-fold CV is not suitable for time series data. It assumes that the observations are independent and identically distributed. However, this assumption is violated when dealing with time series data. Samples in time series data are typically arranged in chronological order.

**Fig. 3:**
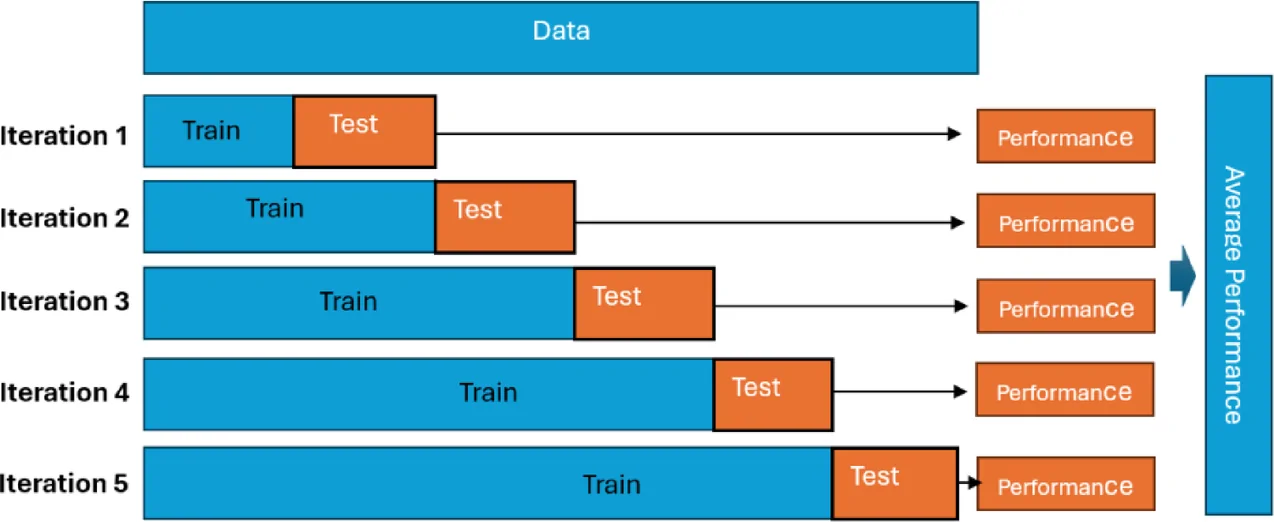
5-fold-TSCV.

TSCV stands for time series cross-validation, an extension of traditional k-fold CV that considers the temporal order of time series data. Unlike traditional cross-validation techniques, which involve randomly shuffling and partitioning data, TSCV preserves the data to ensure that the model is tested on the data that follows the training dataset. TSCV aims to maintain the chronological sequence of observations. In this way, the model is trained on past data and tested on future data^73^. Figure 3 illustrates how TSCV works.

In this study, we used 5-fold-TSCV. The steps of this approach are described as follows:

1. Initial Split Step: The TSCV splits the dataset into 5 blocks. In each block, the observations are arranged in a continuous sequence.
2. Training and Testing Step: In the first iteration, the model is trained on the first block, and the second block is used for model performance evaluation. Similarly, in the second iteration, the first two blocks serve as the training set, and the third block serves as the test set to evaluate the model’s performance.
3. Iterative Process Step: This process is executed 5 times. The TSCV uses an expanding window, where the size of the training set gradually increases with each iteration.
4. Average Results Step: In this phase, the model’s mean across all 5 folds is computed.

### Deep learning models

In this paper, we have employed various recurrent neural network (RNN) variants for stock price prediction, including LSTM, GRU, Bi-LSTM, and Bi-GRU, as well as two hybrid models: GRU-LSTM and CNN-GRU.

LSTM and the GRU are two variants of RNNs. They were developed to solve the vanishing and exploding gradients in RNNs and to process sequential data by incorporating gates and memory cells that can retain previous inputs and efficiently process data^74,75^. The LSTM consists of three gates and a cell state $\:{\mathrm{c}}_{\mathrm{t}}$. These gates are the input gate $\:\:{\mathrm{i}}_{\mathrm{t}\:}$, the forget gate $\:{\mathrm{f}}_{\mathrm{t}\:}$, and the output gate $\:{\mathrm{o}}_{\mathrm{t}\:}$. LSTM can manage the problem of long-term dependencies by integrating these gates^76^. The LSTM model has a complex structure and requires complicated computations. In contrast, the GRU has two gates: an update gate $\:{\mathrm{z}}_{\mathrm{t}\:}$ and a reset gate $\:{\mathrm{r}}_{\mathrm{t}\:}$. Compared with the LSTM model, the GRU accelerates training by combining the input gate and forget gate into a single update gate, using fewer gates and thus reducing the total number of parameters^77,78^. The LSTM and GRU equations are described below:

LSTM:

3 $$ \:{\mathrm{i}}_{{{\mathrm{t}}\:}} = \sigma \:\left( {{\mathrm{w}}_{{\mathrm{i}}} \: \cdot \left[ {{\mathrm{h}}_{{{\mathrm{t}} - 1\:\:}} ,\:{\mathrm{x}}_{{{\mathrm{t}}\:}} } \right] + {\mathrm{b}}_{{\mathrm{i}}} } \right) $$

4 $$\:\:{\mathrm{f}}_{\mathrm{t}\:}={\upsigma\:}\left({\mathrm{w}}_{\mathrm{f}\:}.\left[{\mathrm{h}}_{\mathrm{t}-1\:\:},{\mathrm{x}}_{\mathrm{t}\:}\right]+{\mathrm{b}}_{\mathrm{f}}\right)$$

5 $$\:{\mathrm{o}}_{{{\mathrm{t}}\:}} = \sigma \:\left( {{\mathrm{w}}_{{\mathrm{o}}} \:.\left[ {{\mathrm{h}}_{{{\mathrm{t}} - 1\:\:}} ,{\mathrm{x}}_{{{\mathrm{t}}\:}} } \right] + {\mathrm{b}}_{{\mathrm{o}}} } \right) $$

6 $${\mathrm{Candidate}}\, {\mathrm{value}}\:\stackrel{\sim}{{\mathrm{c}}_{\mathrm{t}}}=\mathrm{t}\mathrm{a}\mathrm{n}\mathrm{h}\left({\mathrm{w}}_{\mathrm{c}}\:.\left[{\mathrm{h}}_{\mathrm{t}-1\:\:},{\mathrm{x}}_{\mathrm{t}\:}\right]+{\mathrm{b}}_{\mathrm{c}}\right)$$

7 $$\:{\mathrm{c}}_{\mathrm{t}}={\mathrm{f}}_{\mathrm{t}\:}\:\mathrm{*}{\mathrm{c}}_{\mathrm{t}-1}+\:{\mathrm{i}}_{\mathrm{t}\:}\mathrm{*}\stackrel{\sim}{{\mathrm{c}}_{\mathrm{t}}}$$

8 $$ {\text{Hidden state h}}_{{\mathrm{t}}} = {\mathrm{o}}_{{{\mathrm{t~}}}} {\mathrm{*}}\tanh {\mathrm{c}}_{{\mathrm{t}}} $$

GRU:

9 $$\:{\mathrm{z}}_{\mathrm{t}\:}={\sigma\:}\left({\mathrm{w}}_{\mathrm{z}}\:\cdot\left[{\mathrm{h}}_{\mathrm{t}-1\:\:},{\:\mathrm{x}}_{\mathrm{t}\:}\right]+{\mathrm{b}}_{\mathrm{z}}\right)$$

10 $$\:\:\:\:\:\:\:{\mathrm{r}}_{\mathrm{t}\:}={\sigma\:}\left({\mathrm{w}}_{\mathrm{r}}\:\cdot\left[{\mathrm{h}}_{\mathrm{t}-1\:\:},{\:\mathrm{x}}_{\mathrm{t}\:}\right]+{\mathrm{b}}_{\mathrm{r}}\right)\:\:\:$$

11 $$ {\text{Candidate hidden state}}~~\widetilde{{{\mathrm{h}}_{{\mathrm{t}}} }} = \tanh ({\mathrm{W}}_{{\mathrm{h}}} \cdot \left[ {{\mathrm{~r}}_{{{\mathrm{t~}}}} {\mathrm{*h}}_{{{\mathrm{t}} - 1}} {\mathrm{~}},{\mathrm{x}}_{{{\mathrm{t~}}}} {\mathrm{~}}} \right] $$

12 $$ {\text{Hidden state}}~{\mathrm{h}}_{{{\mathrm{t~}}}} = \left( {1 - {\mathrm{z}}_{{\mathrm{t}}} } \right){\mathrm{*h}}_{{{\mathrm{t}} - 1}} + \widetilde{{{\mathrm{h}}_{{\mathrm{t}}} }} $$

where $\:{\:\:\mathrm{x}}_{\mathrm{t}\:}$ is the input vector; $\:{\upsigma\:}$ is the sigmoid activation; $\:{{\mathrm{w}}_{\mathrm{i}}\:,\mathrm{w}}_{\mathrm{o}}{\:,\mathrm{w}}_{\mathrm{f}\:},{\mathrm{w}}_{\mathrm{c}}{\:,\mathrm{w}}_{\mathrm{z}}{\:,\mathrm{w}}_{\mathrm{r}},\:{\mathrm{w}}_{\mathrm{h}}:$ is the weight matrix; $\:{{\mathrm{b}}_{\mathrm{i}}\:,\mathrm{b}}_{\mathrm{o}}{\:,\mathrm{b}}_{\mathrm{f}\:},{\mathrm{b}}_{\mathrm{c}}{\:,\mathrm{b}}_{\mathrm{z}}{\:,\mathrm{b}}_{\mathrm{r}}:$ is the bias; $\:{\mathrm{h}}_{\mathrm{t}-1\:\:}:$ is the previous hidden state; and $\:{\mathrm{c}}_{\mathrm{t}-1}:$ is the previous cell state.

The bidirectional RNN consists of two distinct neural networks. Neural networks interpret the data sequence in two opposite directions: forward and backward. Bi-LSTM and Bi-GRU are types of RNN architecture. In Bi-LSTM and Bi-GRU, input information is transmitted in two directions: the forward path, which is from the past to the future, and the backward path, which is from the future to the past. This allows the Bi-LSTM and Bi-GRU to capture and store information from both directions. However, they require more time to train than LSTM or GRU models do^41,68^. The outputs of the Bi-LSTM and Bi-GRU can be calculated as follows:

13 $$ {\text{Output y}} = {\mathrm{g~}}\left( {{\mathrm{w}}_{{\mathrm{y}}} \cdot {\mathrm{~}}\left[ {{\mathrm{a}}_{{{\mathrm{f~~}}}} ,{\mathrm{a}}_{{\mathrm{b}}} } \right]{\mathrm{~}} + {\mathrm{b}}_{{\mathrm{y}}} } \right) $$

where $\:{\mathrm{w}}_{\mathrm{y}}:$ represents weights, $\:{\mathrm{b}}_{\mathrm{y}}:$ represents bias, and $\:{\mathrm{a}}_{\mathrm{f}\:\:},{\mathrm{a}}_{\mathrm{b}}$ represents activation in the forward and backward directions.

A CNN is a type of feedforward neural network. It stands for the Convolutional Neural Network, which is mainly used for feature extraction and processing input data. The structure of a CNN comprises three layers: convolutional, pooling, and fully connected layers^79^. CNNs have achieved remarkable results in image recognition and other fields, such as time series forecasting. When applied to stock price data, they can detect hidden patterns in short-term price fluctuations, such as volatility and trends. However, the CNN model struggles to handle long-term dependencies^80^. A 1D-CNN is used to process sequential data^81^.

### Performance evaluation

We used two complementary evaluation metrics to assess the efficiency and robustness of the proposed models:

1. Standard forecasting accuracy metrics: They measure the predictive accuracy via MSE, MAE, RMSE, and DA.
2. Economic evaluation: It examines the practical forecast value by trading simulation metrics, including strategy return, the strategy Sharpe ratio, and a comparison against a buy-and-hold benchmark for each model.

The formulas for these metrics are presented below:

14 $$ \:MSE{\text{ }} = \frac{1}{n}\sum {\:_{{i = 1}}^{n} } (Y_{i} - \hat{Y}_{i} )^{2} $$

15 $$ MAE{\text{ }} = \:\frac{1}{n}\sum {\:_{{i = 1}}^{n} } \left| {Y_{i} - \hat{Y}_{i} } \right| $$

16 $$ RMSE = \sqrt {MSE} $$

17 $$\:D A = \frac{1}{n}~~\mathop \sum \limits_{{i = 1}}^{n} {\rm I}~\sin \left( {Y_{{i~}} - Y_{{i - 1}} } \right) = = \sin \left( {\hat{Y}_{i} ~ - ~\hat{Y}_{{i - 1}} } \right) $$

where $\:{Y}_{i}$ represents the real value, $\:{\widehat{Y}}_{i}$ represents the predicted value, * n* represents the total number of records, $\:{\rm\:I}$ is the indicator function, and * sin* is the sign function.

18 $$ r_{t}^{{strategy}} = position_{t} ~ \times r_{t} ~~~,~t = 1,2, \ldots ,T $$

$$\:{position}_{t}=1\:if\:f\widehat{{r}_{t}}\:$$ > 0, otherwise, = 0 (19)

20 $$ r_{t}^{{Buy~\& ~hold}} = 1~ \times r_{t} $$

21 $$ V_{T} = \exp ~\left( {\mathop \sum \limits_{{t = 1}}^{T} ~r_{t}^{{strategy~~}} } \right) $$

22 $$\:Total~Return~\left( \% \right) = \left( {\frac{{V_{{T~}} - V_{0} }}{{V_{0} }}} \right)~ \times 100 $$

23 $$ Sharp~Ratio = \frac{{\bar{r}^{{strategy}} }}{{\sigma \left( {r^{{strategy}} } \right)}}~ \times ~\sqrt {252} $$

where $\:{r}_{t}$ represents the actual log return, $\:\widehat{{r}_{t}}\:$ represents the predicted log return, $\:{r}_{t}^{strategy\:\:}$, $\:{r}_{t}^{Buy\:\&\:hold\:\:}$are the daily returns on day$\:\:t$ for the strategy and buy & hold benchmark, respectively, $\:{V}_{0\:}$ and $\:{V}_{T}$ are the starting and ending values of the portfolio over the holdout period, $\:T\:$represents the total number of trading days, $\:{\stackrel{-}{r}}^{strategy}$ and $\:\sigma\:\left({r}^{strategy}\right)$ are the average and standard deviation of daily strategy returns, respectively; the risk-free rate is zero; and $\:\sqrt{252}$ annualizes the Sharpe ratio.

Both the trading strategy and the buy-and-hold benchmark use the same formulas (Eqs. 18, 20–23). The sole difference is that the trading strategy determines the $\:{position}_{t}$ by using the model’s predicted return, whereas the buy-and-hold benchmark assumes$\:{\:position}_{t}=1\:for\:all\:t$.

## Results and discussion

As mentioned above, we conducted our study across different stocks, including AAPL, GS, Pfizer, Ford, and XOM, to measure the efficiency of the proposed models. We train all the DL models in both scenarios, with and without the FS process, to assess the impact of the FS-based models on the prediction results. The results are discussed in the following subsections using different evaluation metrics.

### Predictive performance metrics

Table 4 presents the efficiency of the proposed models with and without the FS process across all the models and stocks using standard forecasting accuracy measures (RMSE, MAE, and DA%). However, these metrics alone are not sufficient for measuring the economic value of the proposed models. In the following section, a complete trading simulation and economic significance analysis are presented as part of the model’s evaluation.

**Table 4:** Forecasting accuracy results of the proposed models.

Model	Stock	Before FS			After FS		
		MAE	RMSE	DA %	MAE	RMSE	DA %
GRU	AAPL	0.0197	0.0256	59.548	0.0180	0.0235	60.54
LSTM		0.0182	0.0241	61.06	0.0159	0.0211	58.80
Bi-GRU		0.0152	0.0198	55.897	0.0149	0.0191	58.97
Bi-LSTM		0.0158	0.0202	57.315	0.0151	0.0199	57.48
GRU_LSTM		0.0175	0.0224	61.790	0.0161	0.0209	59.55
CNN_GRU		0.0176	0.0227	52.6678	0.0156	0.0203	54.39
GRU	Ford	0.0272	0.0358	64.5211	0.0268	0.0350	65.804
LSTM		0.0286	0.0365	62.3167	0.0274	0.0355	63.097
Bi-GRU		0.0226	0.0288	56.9141	0.0223	0.0299	57.7586
Bi-LSTM		0.0230	0.0289	58.3697	0.0221	0.0281	59.9138
GRU_LSTM		0.0248	0.0325	63.3188	0.0223	0.0298	60.2011
CNN_GRU		0.0229	0.0309	52.6393	0.0220	0.0297	51.9537
GRU	GS	0.0219	0.0268	62.015	0.0194	0.0249	62.343
LSTM		0.0178	0.0233	61.5063	0.0183	0.0245	62.5523
Bi-GRU		0.0154	0.0199	52.8616	0.0152	0.0194	56.191
Bi-LSTM		0.0159	0.0201	56.9199	0.0154	0.0201	58.272
GRU_LSTM		0.0169	0.0208	58.7929	0.0163	0.0201	60.353
CNN_GRU		0.0186	0.024	53.7844	0.0183	0.0233	54.548
GRU	Pfizer	0.0143	0.0192	51.9618	0.0139	0.0185	59.408
LSTM		0.0175	0.0231	60.6575	0.0161	0.0216	62.473
Bi-GRU		0.0146	0.0195	55.6139	0.0147	0.0194	58.592
Bi-LSTM		0.0146	0.0194	59.2865	0.0142	0.0199	59.899
GRU_LSTM		0.0168	0.0219	60.3725	0.0147	0.0197	58.281
CNN_GRU		0.0161	0.0212	51.2067	0.0147	0.0192	52.795
GRU	XOM	0.0208	0.0278	62.093	0.0198	0.0258	61.597
LSTM		0.0213	0.0276	60.5619	0.0205	0.0269	61.665
Bi-GRU		0.0176	0.0229	58.7125	0.0173	0.0224	58.982
Bi-LSTM		0.0181	0.0236	60.8512	0.0178	0.0231	57.943
GRU_LSTM		0.0251	0.0256	63.0250	0.0195	0.0254	62.997
CNN_GRU	XOM	0.0187	0.0241	54.2144	0.0185	0.0241	53.974

For Appl. Inc., the Bi-GRU produced the lowest RMSE and MAE after the FS process (0.0191 and 0.0149, respectively). The FS process has a different effect on the DA metric; it increased the DA for the Bi-GRU (55.8 to 58.9) and CNN-GRU (52.6 to 54.4), and it decreased slightly for the LSTM and GRU-LSTM models. Overall, all the proposed models yielded consistent and significant results after the FS process (RMSE and MAE).

For Ford, the GRU reached the highest DA (65.80%), while the Bi-GRU and Bi-LSTM demonstrated the lowest error metrics. Additionally, the FS process has different effects; some models improved, whereas others slightly worsened, which is consistent with the higher return volatility of the Ford company.

For GS, the Bi-GRU produced the lowest RMSE (0.0199 before the FS and 0.0194 after the FS). The FS process led to a consistent increase in the DA metric for all stocks, but with small improvements in the RMSE and MSE.

For Pfizer, the GRU achieved the lowest RMSE and MSE values. The FS process substantially increased the DA for most models. The LSTM model achieved the highest DA (60.65% before the FS and 62.47% after the FS), with a reduction in the RMSE (0.0231 to 0.0216). However, FS negatively impacted the GRU-LSTM model. It has a higher DA before FS (60.37%) but decreases after FS (58.28%), which shows that FS does not benefit all architectures for this stock.

For XOM, Bi-GRU had the lowest RMSE (0.0229 before FS and 0.0224 after FS) and MAE. GRU_LSTM achieved the highest DA (63.02%). The FS process yielded slight but consistent enhancements across the RMSE and MAE metrics, while it had a limited impact on DA, showing a slight decline after the FS across most models. LSTM was the only model to achieve improvements (DA: 60.5% to 61.5%).

Overall, the experimental results confirmed that the Bi-GRU model consistently produced lower RMSEs and MAEs across most stocks. Additionally, the FS process does not always lead to uniform improvements in all the metrics. In some cases, FS reduces the RMSE and MAE, but the DA slightly decreases, as the goal of FS is mainly to reduce error and not improve directional prediction, indicating the importance of measuring the models across different metrics.

### Trading analysis and economic significance evaluation

The trading performance of each model is classified into three categories related to trading verdict: strong (outperforms buy-and-hold benchmark on both return and Sharpe ratio), weak (outperforms on return only), and poor (fails to outperform the buy-and-hold benchmark).

A strong verdict indicates relative outperformance against the buy-and-hold benchmark and does not necessarily mean positive absolute profitability. Additionally, Table 5 presents the profitability analysis of the proposed models with and without the FS process across all stocks using economic evaluation metrics.

**Table 5:** Trading simulation results of the proposed models against the buy-and-hold strategy.

Model	Stock	Strategy Return % (Before FS)	Strategy Return % (After FS)	Strategy Sharpe (Before FS)	Strategy Sharpe (After FS)	Trading Verdict	Beats Buy-Hold Benchmark
Buy & Hold	AAPL	12.947	12.947	0.1892	0.1892	-	-
GRU		60.57	83.39	1.0566	1.4295	Strong	TRUE
LSTM		68.80	73.76	1.1875	1.2735	Strong	TRUE
Bi-GRU		72.828	90.651	1.2431	1.3487	Strong	TRUE
Bi-LSTM		51.515	85.756	0.9089	1.3052	Strong	TRUE
GRU-LSTM		68.44	89.106	1.2135	1.3837	Strong	TRUE
CNN-GRU		20.15	75.838	0.4368	1.2438	Strong	TRUE
Buy & Hold	Ford	2.399	2.399	0.0206	0.0206	-	-
GRU		24.996	39.6512	0.2569	0.3787	Strong	TRUE
LSTM		45.347	53.128	0.4377	0.522	Strong	TRUE
Bi-GRU		25.911	72.987	0.24530	0.6412	Strong	TRUE
Bi-LSTM		15.127	24.832	0.1528	0.2683	Strong	TRUE
GRU-LSTM		42.090	54.301	0.4380	0.5044	Strong	TRUE
CNN-GRU		33.719	40.85	0.3134	0.3968	Strong	TRUE
Buy & Hold	GS	150.017	150.017	0.9242	0.9242	-	-
GRU		36.2629	46.7547	0.4665	0.5890	Poor	FALSE
LSTM		28.774	49.488	0.3428	0.5767	Poor	FALSE
Bi-GRU		60.456	70.987	0.4521	0.6975	Poor	FALSE
Bi-LSTM		38.15	44.818	0.4913	0.5526	Poor	FALSE
GRU-LSTM		23.19	46.545	0.2821	0.5256	Poor	FALSE
CNN-GRU		36.23	63.996	0.4355	0.6495	Poor	FALSE
Buy & Hold	Pfizer	-8.734	-8.734	-0.0937	-0.0937	-	-
GRU		16.39	30.325	0.2103	0.3896	Strong	TRUE
LSTM		25.78	45.738	0.3381	0.5809	Strong	TRUE
Bi-GRU		29.687	49.793	0.3607	0.5805	Strong	TRUE
Bi-LSTM		9.78	12.058	0.1287	0.1779	Strong	TRUE
GRU-LSTM		23.92	38.556	0.2961	0.5148	Strong	TRUE
CNN-GRU		9.68	14.895	0.1452	0.1896	Strong	TRUE
Buy & Hold	XOM	239.775	239.775	1.0486	1.0486	-	-
GRU		87.91	169.73	0.8152	1.3316	Poor	FALSE
LSTM		78.75	91.02	0.7378	0.7768	Poor	FALSE
Bi-GRU		129.77	163.96	0.8975	0.9654	Poor	FALSE
Bi-LSTM		56.29	104.23	0.5382	0.8681	Poor	FALSE
GRU-LSTM		43.38	94.50	0.5032	0.8297	Poor	FALSE
CNN-GRU		49.55	85.56	0.4808	0.7238	Poor	FALSE

The impact of the FS process on trading profitability was asset-specific for each stock rather than uniform across all stocks. AAPL, Ford, and Pfizer showed strong trading performance. All the proposed models consistently exceeded the buy-and-hold benchmark. In contrast, for GS and XOM, none of the proposed models could beat the buy-and-hold strategy and reported poor verdicts across all the models because of high returns for the buy-and-hold strategy from sustained strong bullish trend markets during the holdout period, reflecting market conditions.

For AAPL, the FS process had a significant effect on the strategy returns for the Bi-GRU model, increasing from 72.82% to 90.65%. This led to a major improvement in the CNN-GRU (20.15% to 75.83%), which was the worst model before the FS. For Ford, the Bi-GRU had the highest strategy returns after the FS (72.9%). Additionally, the FS process had a significant positive effect on different modes, such as the Bi-GRU (25.9% to 72.9%), GRU (24.996% to 39.6512%), and Bi-LSTM (15.12% to 24.83%). In addition to Pfizer, Bi-GRU had the highest return strategy (49.79%). The FS process consistently enhanced returns across all the models; the LSTM process improved the most (25.78% to 45.73%). Additionally, it has a significant effect on the GRU-LSTM model, increasing from 23.92% to 38.55%. We observed similar improvements in risk-adjusted performance before and after the FS process across all stocks and models.

**Fig. 4:**
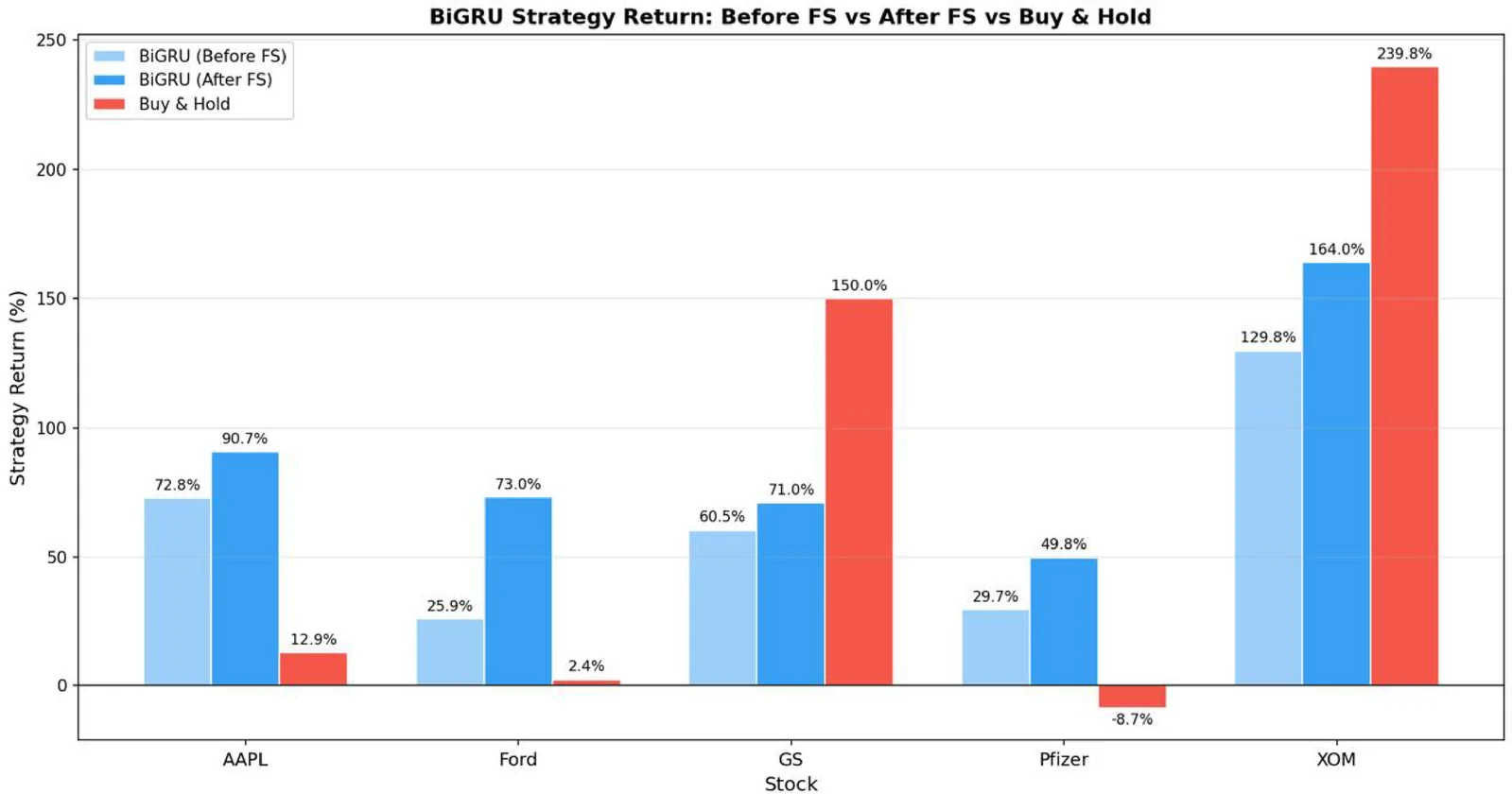
Bi-GRU strategy returns vs. buy-and-hold benchmark across all stocks.

The strategy returns of the best-performing model (Bi-GRU) before and after the FS process against the buy-and-hold benchmark across all stocks are presented in Fig. 4, showing a clear visual of how the proposed hybrid FS process has led to a higher trading profitability across all stocks.

Overall, the profitability of trading depends only on whether the predicted return is positive or negative. The FS process can reduce sign errors by eliminating noisy features that directly increase trading profits but only have a marginal effect on the RMSE and MAE. The results revealed that the FS process consistently enhanced trading performance across all stocks and models.

To ensure a rigorous and unbiased evaluation environment, the sample covers stocks across different markets during the test period: strongly bullish (XOM: +239% and GS: +150%), moderate (AAPL: +13% and Ford: +2.399%), and declining (Pfizer: -9%) markets. For risk-averse investors in declining markets, a strong verdict is valuable, as it means that the model limits.

losses compared to the buy-and-hold strategy (passive holding).

#### Statistical hypothesis test results

In this study, we employed different statistical tests, including the Wilcoxon signed-rank test^82^, the Friedman test^83^, the Finner post hoc test^84^, and the DM test^85^, to ensure the reliability and robustness of the results. We performed these tests in four stages:

#### Impact of the FS process

We conducted the Wilcoxon signed-rank test to compare the paired results obtained from each model’s performance before and after using the FS mechanism. We used this test to evaluate the statistical significance of the FS process. The null hypothesis of this test is that the differences between the two groups (without FS vs. with FS) are statistically equal.

The findings of this test are presented in Table 6. With respect to the results, the null hypothesis is rejected (p-value < 0.001). The results indicated that the FS-based framework significantly enhances model performance and consistently achieves superior results across the RMSE, MAE, strategy return, and Sharpe ratio.

**Table 6:** Wilcoxon signed-rank test results.

*P* value				Significant Level (0.05)	Interpretation
RMSE	MAE	Strategy Total Return %	Strategy Sharpe		
< 0.001	< 0.001	< 0.001	< 0.001	**Significant**	The FS-based framework significantly outperforms

#### Model comparison

We applied the Friedman test to the obtained results to determine whether the differences between the proposed models are statistically significant. The null hypothesis of the Friedman test is that the means of the proposed models are equal.

The findings of this test are summarized in Table 7. With respect to the findings, the null hypothesis is rejected (p-value < 0.05) across RMSE and MAE, and the proposed models are ranked from best to worst (lowest to highest). The results revealed that the Bi-GRU model outperforms the other models, achieving superior performance across different metrics.

**Table 7:** Friedman and Finner post hoc test results.

Metric	Model	Mean Ranking	Friedman *P*-value	Adjusted *P*-value (Finner)	Significant
RMSE	**Bi-GRU**	**1.917**	0.0025	**-**	**-**
	Bi-LSTM	2.000		1	No
	CNN-GRU	3.167		0.37471	No
	GRU-LSTM	3.583		0.53686	No
	GRU	4.833		0.02709	**Yes**
	LSTM	5.500		0.00595	**Yes**
MAE	**Bi-GRU**	**1.833**	0.0024	**-**	**-**
	Bi-LSTM	1.917		1	No
	GRU-LSTM	3.417		0.48068	No
	CNN-GRU	3.667		0.75817	No
	GRU	4.833		0.03416	**Yes**
	LSTM	5.333		0.00453	**Yes**

### Post hoc analysis

We subsequently employed the Finner post hoc test to conduct pairwise comparisons using the Bi-GRU model as the control method. The null hypothesis of this test is that the mean difference between the control method (Bi-GRU) and the other models is equal.

The findings of this test are presented in Table 7. The null hypothesis is rejected (p-value < 0.05) in some cases when the Bi-GRU model is compared with the other models.

The findings confirm that the Bi-GRU model achieves significant superiority over the LSTM and GRU models across the RMSE and MAE metrics. In contrast, we observe that the differences between the Bi-GRU and other models (Bi-LSTM, GRU-LSTM, and CNN-GRU) are equal.

### Forecast accuracy comparison

The DM test is applied to statistically examine the forecasting accuracy of the top-performing model (Bi-GRU) with standard forecasting models (LR and RF). Table 8 shows the performance of this test in terms of the RMSE and MAE metrics.

**Table 8:** DM test results for the Bi-GRU against benchmark models.

Company	Benchmark Model	DM Statistic RMSE	DM Statistic MAE
AAPL	LR	5.0806***	6.5891***
AAPL	RF	5.5474***	7.0198***
Ford	LR	5.0472***	6.3707***
Ford	RF	4.8448***	6.2109***
GS	LR	9.2357***	9.9045***
GS	RF	9.6213***	10.519***
Pfizer	LR	7.009***	8.2928***
Pfizer	RF	7.0899***	8.4582***
XOM	LR	8.2374***	9.0428***
XOM	RF	8.3252***	9.1527***

### Comparative analysis with conventional ML models

To measure the significance of the proposed framework, we benchmarked the top-performing model (Bi-GRU) against standard forecasting models (LR and RF) using different statistical and economic evaluation metrics. Table 9 summarizes this comparison across all stocks. The results of the buy-and-hold benchmark and the Bi-GRU model are reported above in Tables 4 and 5.

**Table 9:** Performances of the LR and RF models across all stocks.

Company	Model	RMSE	MAE	DA	Strategy Return	Sharpe Ratio	Trading Verdict
AAPL	LR	0.0178	0.0131	45.178	66.036	0.9859	Strong
	RF	0.0176	0.0130	41.624	71.592	0.8773	Strong
Ford	LR	0.0263	0.0192	40.51	-8.893	-0.1203	Poor
	RF	0.0264	0.0192	39.518	8.801	0.1044	Strong
GS	LR	0.0166	0.0127	54.48	-89.53	-3.1592	Poor
	RF	0.0163	0.0125	43.563	39.124	0.3578	Poor
Pfizer	LR	0.0162	0.0118	48.566	-54.841	-1.1746	Poor
	RF	0.0161	0.0117	47.541	-66.073	-1.4184	Poor
XOM	LR	0.0195	0.0148	55.898	-93.371	-3.7991	Poor
	RF	0.0194	0.0147	36.588	-87.756	-2.3543	Poor

Table 9 shows that, compared with the results of the Bi-GRU model in Table 4, the LR and RF models produced lower values of the prediction accuracy metrics (RMSE and MAE) across all the stocks, whereas the DA metric reveals that the Bi-GRU model consistently outperforms the LR and RF models across all stocks.

To determine if the differences are statistically significant, we applied the DM test using the RMSE and MAE metrics. According to the results in Table 8, the positive DM statistic indicates that compared with Bi-GRU, LR and RF have significantly lower forecast errors for both the RMSE and the MAE (P value < 0.001).

**Fig. 5:**
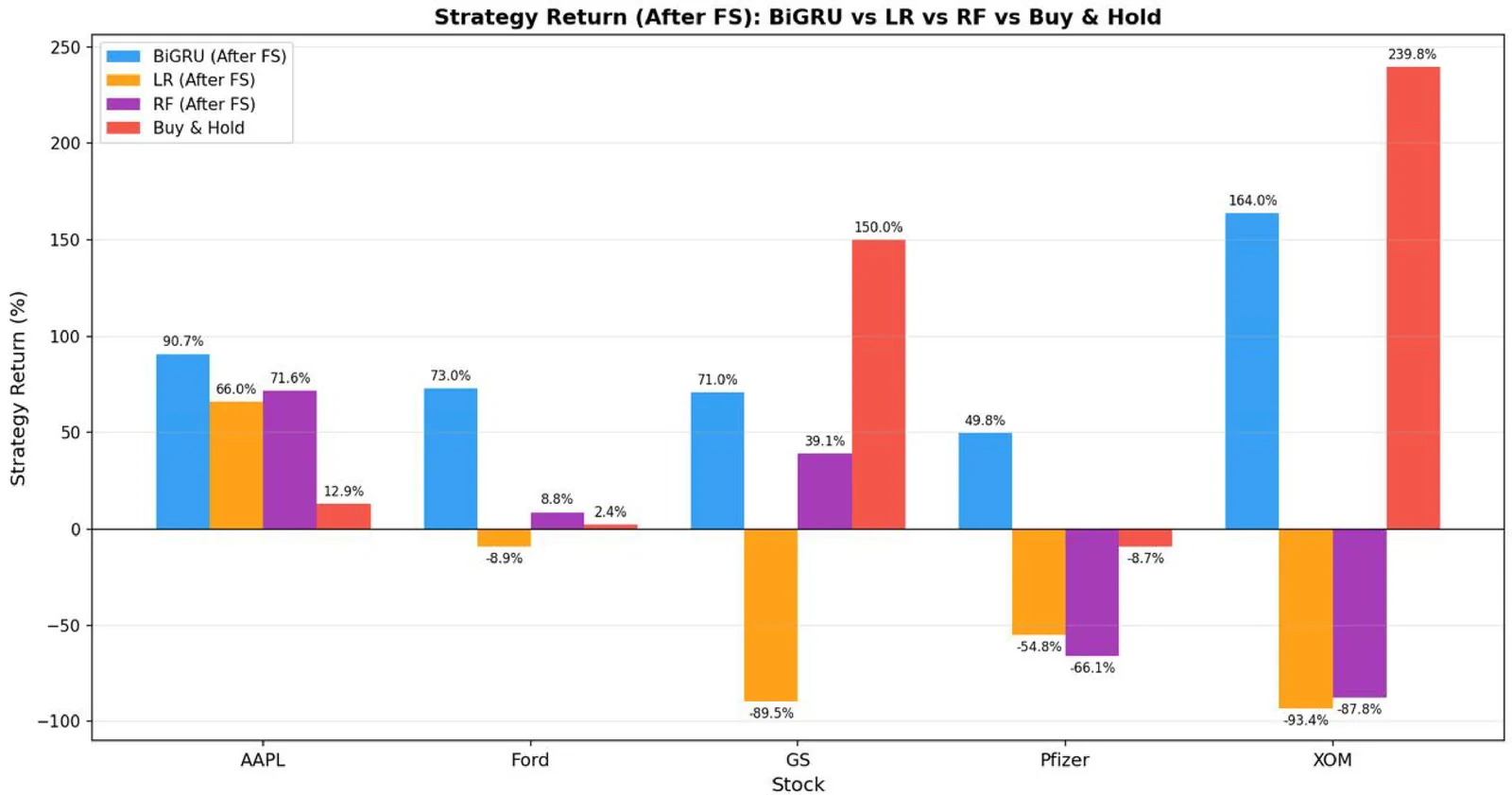
Strategy returns of Bi-GRU, LR, RF vs. buy-and-hold benchmark across all stocks.

The strategy returns of the Bi-GRU, LR, and RF vs. the buy-and-hold benchmark across all stocks after the FS process are shown in Fig. 5, which reveals that the trading profitability of the Bi-GRU is higher than that of traditional models.

Although the LR and RF models outperform the Bi-GRU model in terms of the RMSE and MAE, their trading performance and economic significance are substantially inferior. They recorded poor trading verdicts in 7 out of 10 cases and severe losses in many stocks (LR: -89.53% for GS and − 93.37% for XOM), as described in Table 9, while Bi-GRU produced positive and higher returns with superior risk-adjusted performance across all stocks, as illustrated in Fig. 5. This confirms that lower forecasting error does not necessarily translate to higher trading performance, supporting the usage of DL models in the applications of financial trading.

### Ablation study

An ablation study was conducted on the Bi-GRU model to comprehensively evaluate the contribution of each significant component within the framework, as it achieved higher trading returns and superior economic performance across all companies. We reported the results of this study as averages over all stocks, providing a more representative assessment of each component’s contribution. Since FS had a consistently significant effect across all stocks on the trading simulation metrics, more than the forecasting accuracy metrics (RMSE and MAE), this study focused on the strategy return and Sharpe ratio, reflecting the added economic value from each component.

Three configurations were compared: (Baseline): using OHLCV features and TIs; (Extended): using OHLCV features and TIs combined with macroeconomic data and volatility features; and (Proposed): using all features (OHLCV, TIs, macroeconomic data) along with the hybrid FS approach. The findings of the ablation study are presented in Table 10.

**Table 10:** Ablation study results: contribution of each component within the proposed work.

Configuration	Features	FS	Avg Strategy Return_%	Avg Strategy Sharpe
Baseline	H + T	No	50.5902	0.3913
Extended	H + T+M	No	69.1627	0.6812
Proposed	H + T+M + FS	Yes	89.2776	0.8647

H: Historical data, T: Technical indicators, M: Macroeconomic data, FS: Feature selection.

As shown in Table 10, excluding macroeconomic data and the FS process leads to lower economic performance across all trading metrics. The results revealed that adding macroeconomic data and volatility features to TIs achieved significant improvements (strategy return: 50.59% to 69.16%, Sharp: 0.391 to 0.681), whereas the incorporation of the FS process resulted in the most substantial gains (strategy return: 89.277%, Sharp: 0.864).

A comparative analysis revealed that each component significantly enhanced trading performance. The complete model, combining all the features and the FS process, achieved superior performance and produced higher trading returns, reflecting the complementary contributions of all the features. These results confirm that the FS process is the most influential component and contributes mainly to economic performance, which is consistent with the results of the Wilcoxon test.

### SHAP interpretability

**Fig. 6:**
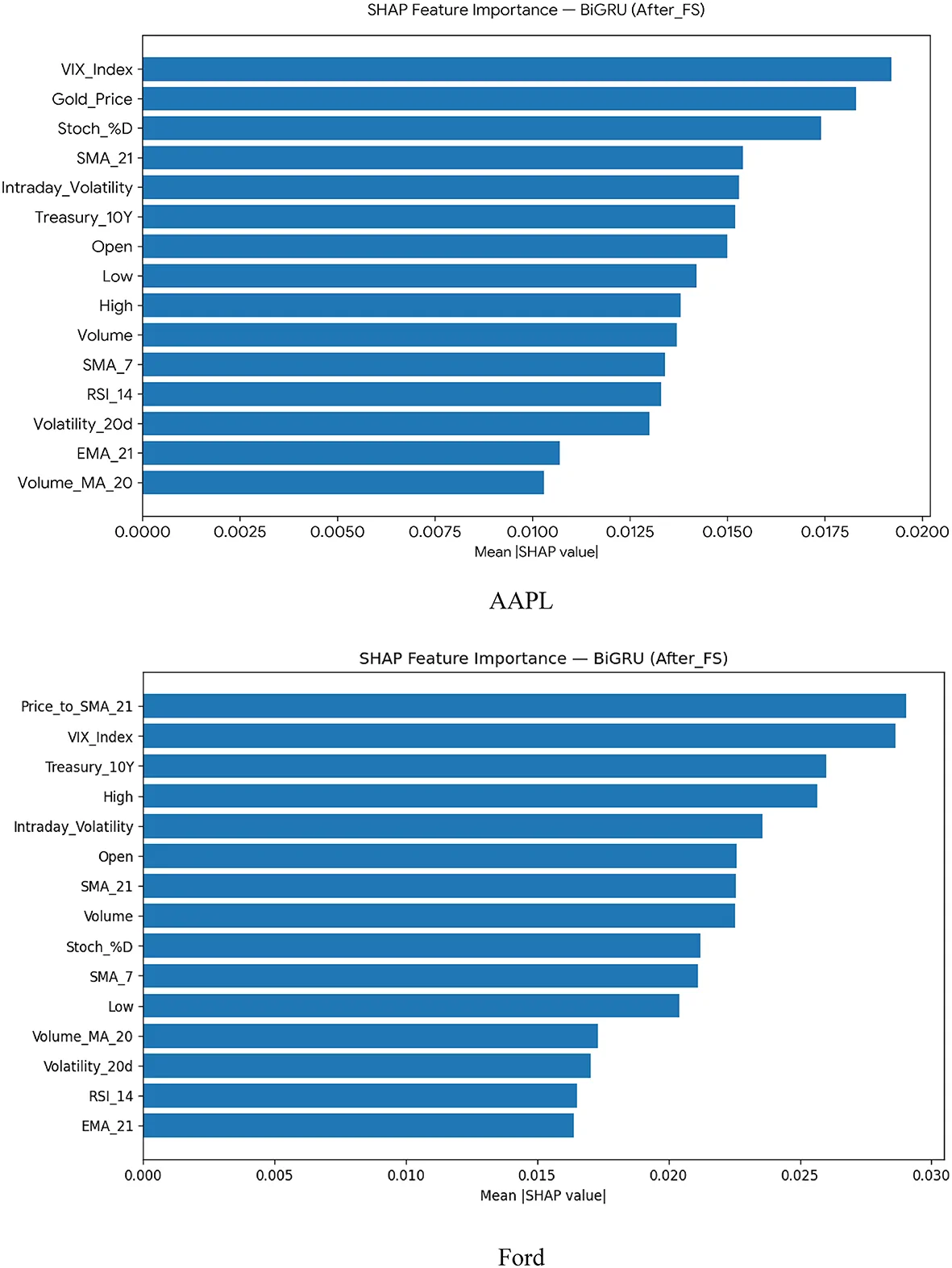

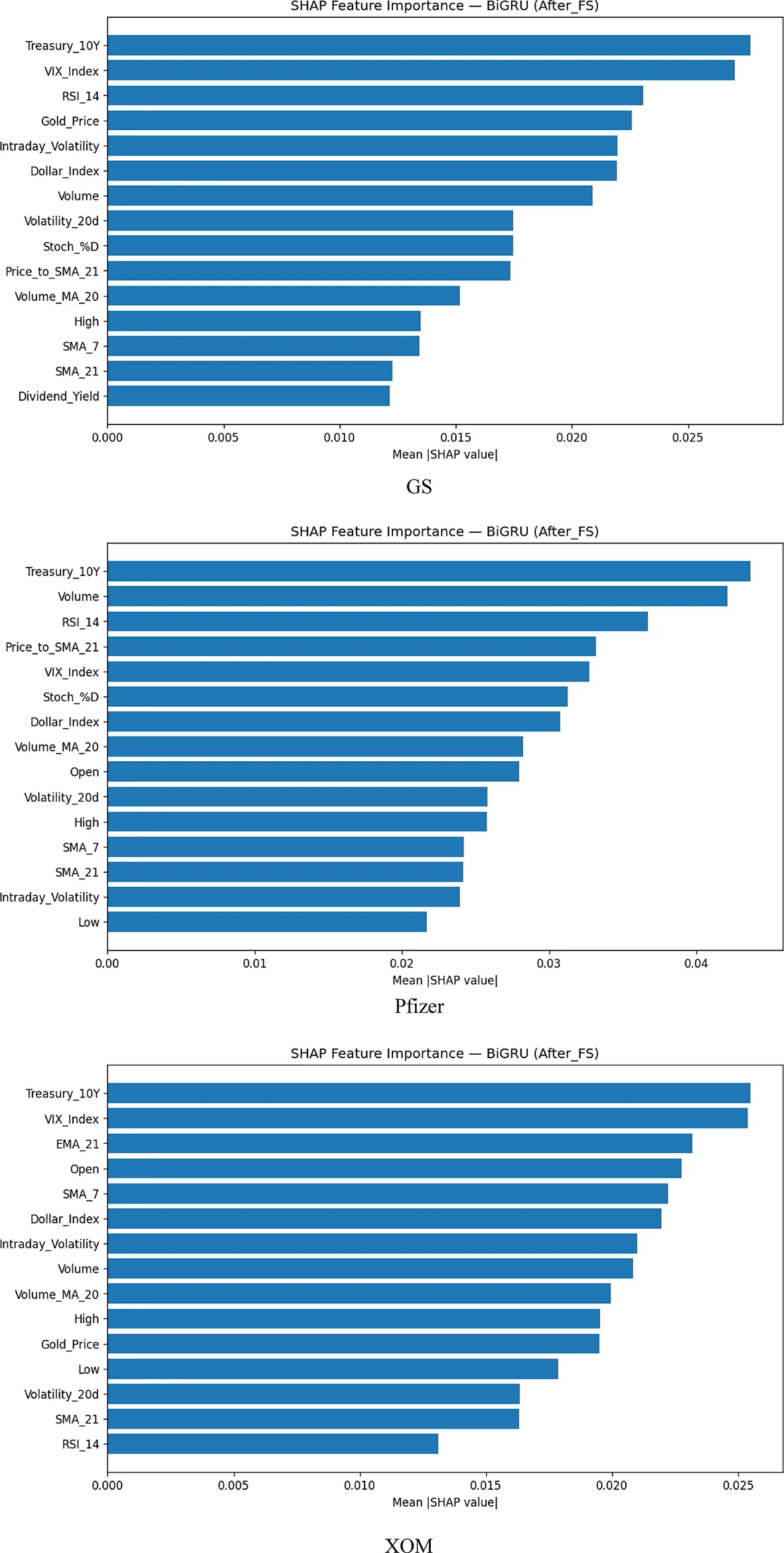
SHAP features importance: bar plot for all stocks.

SHAP is an explainable artificial intelligence (XAI) technique that allows the complex decisions of deep models to be understood. It was introduced by^86^ to explain individual predictions. Deep neural networks are often referred to as black boxes because of their opacity. SHAP explains a model’s predictions by assigning each feature to an interpretable value and offering insight into how each variable individually influences the model’s predictions. The SHAP bar plots of each stock are shown in Fig. 6.

The SHAP analysis shows that both macroeconomic and technical indicators have a major effect on the explanation of stock return dynamics. Treasury-10y and VIX are the most significant macroeconomic factors that influence the predictions of the Bi-GRU across all the stocks, which is consistent with the theory of monetary policy (Treasury_10y) and the risk premium literature (VIX).

The significance of the VIX index is consistent with the risk premium literature, whereas the Treasury yield’s very high predictive role supports asset pricing and monetary policy theories, as it reflects expectations about economic growth and inflation. Gold prices appeared for APPL and GS stocks, indicating the role of safe-haven dynamics during times of uncertainty, whereas stock-specific features, such as the Dollar index for XOM, which indicates oil-dollar dynamics, and Dividend Yield for GS, which is used to understand the sector-specific valuation effects. In addition, the significance of TIs such as moving averages, RSI_14, and stochastic oscillators is consistent with the technical trading and market momentum literature. Overall, these results confirm that the proposed model efficiently combines significant insights from both macroeconomic fundamentals and stock market dynamics.

### Comparison with other approaches

Previous research has focused mainly on the prediction of stock prices. In contrast, the focus of this study is to predict stock returns, so a quantitative comparison between the prediction errors is ineffective and not appropriate. Therefore, the comparison is conducted from a methodological perspective rather than numerical forecasting errors. Factors such as the prediction target, feature set, validation strategy, proposed models, and overall predictive performance are considered for that comparison.

In this section, we consider different baseline models for comparison on the basis of their features. The baseline models are categorized into two types: DL models that use only historical stock price data and DL models that incorporate historical stock price data along with different TIs. A methodological comparison of the proposed framework with those of previous studies is summarized in Table 11, highlighting the main advancements introduced in this study.

**Table 11:** Methodological comparison with previous studies.

Study	Target Variable	Models	FS Applied	Features Used	Validation Approach	Statistical Evaluation	Economic Evaluation
^11^	Stock Price	LSTM	No	H + T	Holdout Split	Yes	No
^13^	Stock Price	LSTM, GRU	No	H	Holdout Split	Yes	No
^30^	Stock Price	ARIMA-RNN	No	H	Holdout Split	Yes	No
^43^	Stock Price	ELM, LSTM, MLP-B, ANN, XGBoost	No	H + T	Holdout Split	Yes	No
^54^	Stock Price	LSTM	Yes (PCA)	H + T	Holdout Split	Yes	No
Proposed	Stock Return	GRU, LSTM, Bi-GRU, Bi-LSTM, GRU-LSTM, CNN-GRU, LR, RF	Yes Hybrid FS via filter and wrapper methods	H + T+V + M	“Hybrid” TSCV+ Holdout	Yes	Yes Strategy Return, Sharpe ratio, buy-and-hold benchmark

H: Historical data, T: Technical indicators, V: Volatility, M: Macroeconomic data.

As shown in Table 11, we conclude that by incorporating various feature engineering methods, hybrid FS processes, robust validation, and comprehensive performance evaluation, including both statistical and economic metrics, the proposed framework offers a more comprehensive methodology than previous methods.

## Conclusion

In this study, a unified framework for predicting daily log returns that incorporates comprehensive data, hybrid FS, economic evaluation, and advanced DL models is developed. The inclusion of TIs, volatility features, and macroeconomic data alongside financial data provides valuable insights into market behavior.

Identifying the most significant features to enhance stock price prediction has become increasingly complex and challenging. In this study, we demonstrated the impact of using the complete feature set on model performance. We showed how to address this issue via a combination of filter and wrapper methods. The efficiency of the proposed models was assessed via different evaluation metrics: statistical evaluation, such as MSE, MAE, RMSE, and DA; and economic evaluation, such as strategy returns, the Sharpe ratio, and a comparison against the buy-and-hold benchmark. All the proposed models were tested across five datasets: AAPL, GS, Ford, PFE, and XOM. The results reveal that combining the filter and wrapper FS methods with DL models yields outstanding results and significantly improves the trading profitability across all the models and stocks.

The findings confirmed that the Bi-GRU model is consistently superior to the other DL models across different stocks and evaluation metrics, indicating its ability to generate reliable and accurate predictions and profitable returns. These findings revealed the potential of combining historical prices, macroeconomic data, and TIs to enhance stock return prediction. According to the results of the Wilcoxon test, the FS-based framework significantly improves model performance across all stocks. The reduction in DA for some models after FS was anticipated since the features were chosen to minimize forecast error (MSE) and not maximize DA. This trade-off is commonly reported in the financial forecasting literature.

To evaluate the significance of the proposed framework, the best-performing model (Bi-GRU) was compared to traditional ML models (LR and RF) and the buy-and-hold strategy. The results of this study demonstrate an important and consistent finding: low forecast error does not necessarily improve trading profitability. Empirical results revealed that although LR and RF achieved the lowest RMSE and MAE performance, Bi-GRU consistently produced the highest economic returns. These results not only question the practice of using statistical accuracy (MSE, RMSE, and MAE) as the main criterion for model evaluation but also highlight economic significance analysis, such as trading returns and Sharpe ratios, as a crucial and complementary performance evaluation framework.

This study not only highlights the benefits of the proposed models in enhancing stock price prediction for understanding the behavior of different stock markets, providing significant insights for investors and financial analysts, but also demonstrates that integrating different features with hybrid FS methods and DL models provides a robust framework for forecasting stock closing prices, improving performance and reliability in predicting future stock prices.

In the future, we intend to incorporate Twitter data and financial news alongside the baseline features and investigate ensemble methods to improve the results. Additionally, other macroeconomic factors, such as EPU, GPR, and the inflation rate, are integrated to evaluate their performance in predicting stock returns.

## Data Availability

The data supporting the findings of this study are available upon request.
